# A Population of Indirect Pathway Striatal Projection Neurons Is Selectively Entrained to Parkinsonian Beta Oscillations

**DOI:** 10.1523/JNEUROSCI.0658-17.2017

**Published:** 2017-10-11

**Authors:** Andrew Sharott, Federica Vinciati, Kouichi C. Nakamura, Peter J. Magill

**Affiliations:** ^1^Medical Research Council Brain Network Dynamics Unit, University of Oxford, Oxford OX1 3TH, United Kingdom, and; ^2^Oxford Parkinson's Disease Centre, University of Oxford, Oxford OX1 3QX, United Kingdom

**Keywords:** basal ganglia, dopamine, electrophysiology, oscillations, Parkinson's disease, striatum

## Abstract

Classical schemes of basal ganglia organization posit that parkinsonian movement difficulties presenting after striatal dopamine depletion stem from the disproportionate firing rates of spiny projection neurons (SPNs) therein. There remains, however, a pressing need to elucidate striatal SPN firing in the context of the synchronized network oscillations that are abnormally exaggerated in cortical–basal ganglia circuits in parkinsonism. To address this, we recorded unit activities in the dorsal striatum of dopamine-intact and dopamine-depleted rats during two brain states, respectively defined by cortical slow-wave activity (SWA) and activation. Dopamine depletion escalated striatal net output but had contrasting effects on “direct pathway” SPNs (dSPNs) and “indirect pathway” SPNs (iSPNs); their firing rates became imbalanced, and they disparately engaged in network oscillations. Disturbed striatal activity dynamics relating to the slow (∼1 Hz) oscillations prevalent during SWA partly generalized to the exaggerated beta-frequency (15–30 Hz) oscillations arising during cortical activation. In both cases, SPNs exhibited higher incidences of phase-locked firing to ongoing cortical oscillations, and SPN ensembles showed higher levels of rhythmic correlated firing, after dopamine depletion. Importantly, in dopamine-depleted striatum, a widespread population of iSPNs, which often displayed excessive firing rates and aberrant phase-locked firing to cortical beta oscillations, preferentially and excessively synchronized their firing at beta frequencies. Conversely, dSPNs were neither hyperactive nor synchronized to a large extent during cortical activation. These data collectively demonstrate a cell type-selective entrainment of SPN firing to parkinsonian beta oscillations. We conclude that a population of overactive, excessively synchronized iSPNs could orchestrate these pathological rhythms in basal ganglia circuits.

**SIGNIFICANCE STATEMENT** Chronic depletion of dopamine from the striatum, a part of the basal ganglia, causes some symptoms of Parkinson's disease. Here, we elucidate how dopamine depletion alters striatal neuron firing *in vivo*, with an emphasis on defining whether and how spiny projection neurons (SPNs) engage in the synchronized beta-frequency (15–30 Hz) oscillations that become pathologically exaggerated throughout basal ganglia circuits in parkinsonism. We discovered that a select population of so-called “indirect pathway” SPNs not only fire at abnormally high rates, but are also particularly prone to being recruited to exaggerated beta oscillations. Our results provide an important link between two complementary theories that explain the presentation of disease symptoms on the basis of changes in firing rate or firing synchronization/rhythmicity.

## Introduction

Chronic depletion of dopamine from dorsal striatum and other basal ganglia (BG) nuclei is thought to underlie bradykinesia and rigidity in idiopathic Parkinson's disease (PD). The influential “direct/indirect pathways” model of BG organization (DeLong, 1990; Smith et al., 1998) posits that dopamine depletion changes the activity of spiny projection neurons (SPNs) in striatum, resulting in a gross imbalance in the firing rates of direct pathway SPNs (dSPNs) and indirect pathway SPNs (iSPNs). Because SPN firing mediates striatal output, this rate imbalance is predicted to have dire consequences for neuronal activity in other BG nuclei and then behavior.

Studies of idiopathic PD and its animal models have advanced the complementary notion that excessive oscillatory synchronization of BG neuronal activity, particularly at beta frequencies (typically defined as 15–30 Hz), underlies bradykinesia/rigidity (Kühn et al., 2006, 2008, 2009; Ray et al., 2008; Sharott et al., 2014). Excessive (parkinsonian) beta oscillations have been observed in the activity of neurons in external globus pallidus (GPe), subthalamic nucleus (STN), and other BG nuclei outside of striatum (Brown et al., 2001; Sharott et al., 2005; Mallet et al., 2008a; Avila et al., 2010); these abnormal temporal dynamics are often, but not always, concomitant with altered firing rates (Mallet et al., 2008a,b; Steigerwald et al., 2008; Sharott et al., 2014; Galvan et al., 2015). Although these BG nuclei are typically conceived to be “downstream” of striatum (Gerfen and Surmeier, 2011), their expression of parkinsonian beta oscillations is not necessarily orchestrated by striatal outputs. Indeed, several reports have instead stressed that a network of reciprocally connected GPe and STN neurons, influenced by direct cortical inputs to STN (potentially bypassing striatum), could generate parkinsonian beta oscillations and, thus, play key roles in propagating these abnormal rhythms throughout cortico-basal ganglia circuits (Holgado et al., 2010; Tachibana et al., 2011; Pavlides et al., 2012; Holt and Netoff, 2014; Ahn et al., 2016). Alternatively, some computational models forecast that parkinsonian beta oscillations originate within networks of striatal neurons, albeit via different mechanisms (McCarthy et al., 2011; Damodaran et al., 2015). Others have argued that, regardless of whether striatum generates beta oscillations, increased striatal output after dopamine depletion is critical for the emergence of these rhythms in the GPe–STN network (Kumar et al., 2011). Further modeling predicts that striatal output is abnormally synchronized at beta frequencies and that this is important for the pathological oscillatory entrainment of GPe neuron activity (Nevado-Holgado et al., 2014; Corbit et al., 2016; Lindahl and Hellgren Kotaleski, 2016). Despite informative work *in silico* and *in vitro*, it is not certain that SPNs *in vivo* synchronize their spike firing at beta frequencies after dopamine depletion. Exaggerated beta oscillations arise in the local field potentials (LFPs) recorded from striatum during activated brain states in anesthetized dopamine-depleted rats (Moran et al., 2011). However, SPNs have been reported to be “silent” under similar circumstances (Mallet et al., 2006). Thus, exaggerated beta oscillations in striatal LFPs might not be accompanied by excessively synchronized SPN spike firing at beta frequencies. There are clear precedents for dissociations between striatal LFP oscillations and striatal neuron firing in pathophysiological states. For example, in a rat model of absence epilepsy, pathological spike-and-wave oscillations at 7–10 Hz are readily detected in striatal LFPs, and yet, SPNs do not discharge spikes during these highly synchronous network events (Slaght et al., 2004).

Resolving whether and how striatum is engaged by parkinsonian beta oscillations in cortical–basal ganglia circuits requires definitions of SPN spike firing *in vivo*. To address this, we quantified the brain state-dependent activity of single neurons and larger neuronal populations recorded in striatum of anesthetized dopamine-intact and dopamine-depleted rats. Data were interpreted in light of the firing of identified dSPNs and iSPNs recorded under the same conditions. Our results emphasize the potential importance of an aberrant, selective entrainment of the firing of a population of iSPNs.

## Materials and Methods

All experimental procedures were performed on adult male Sprague Dawley rats (Charles River) and were conducted in accordance with the Animals (Scientific Procedures) Act, 1986 (United Kingdom). All experimental work adhered to the Society for Neuroscience Policies on the Use of Animals in Neuroscience Research.

### 

#### 

##### 6-Hydroxydopamine lesions of midbrain dopamine neurons.

Unilateral 6-hydroxydopamine (6-OHDA) lesions were induced in rats weighing 190–280 g, as previously detailed (Mallet et al., 2008a,b, 2012; Abdi et al., 2015). Briefly, the neurotoxin 6-OHDA (hydrochloride salt; Sigma-Aldrich) was dissolved in 0.9% w/v ice-cold NaCl solution containing 0.02% w/v ascorbate to a final concentration of 12 mg/ml. Approximately 25 min before the injection of 6-OHDA, all animals received desipramine (25 mg/kg, i.p.; Sigma-Aldrich) to minimize the uptake of 6-OHDA by noradrenergic neurons. Anesthesia was induced and maintained with 1.5–3% v/v isoflurane in O_2_, and animals were placed in a stereotaxic frame (Kopf). Body temperature was maintained at 37 ± 0.5°C by a homeothermic heating device (Harvard Apparatus). Under stereotaxic control, 1 μl of 6-OHDA solution was injected near the medial forebrain bundle (4.1 mm posterior and 1.2–1.4 mm lateral of Bregma, and 7.9 mm ventral to the dura; Paxinos and Watson, 2007). Lesions were assessed 14 or 15 d after 6-OHDA injection by challenge with apomorphine (0.05 mg/kg, s.c.; Sigma-Aldrich; Schwarting and Huston, 1996) and were considered successful when animals made ≥80 net contraversive rotations in 20 min (Abdi et al., 2015). Electrophysiological recordings (see below) were performed in the dorsal striatum ipsilateral to 6-OHDA lesions in anesthetized rats 21–39 d after surgery.

##### *In vivo* electrophysiological recording and juxtacellular labeling of individual striatal neurons.

Recording and labeling experiments were performed in 36 anesthetized control rats (age, 3–4 months; weight, 295–390 g) and 17 anesthetized 6-OHDA-lesioned rats (age, 3–5 months; weight, 305–430 g at the time of recording), as previously described (Mallet et al., 2008a,b, 2012). Briefly, anesthesia was induced with 4% v/v isoflurane in O_2_, and was maintained with urethane (1.3 g/kg, i.p.; ethyl carbamate, Sigma-Aldrich) and supplemental doses of ketamine (30 mg/kg, i.p.; Willows Francis) and xylazine (3 mg/kg, i.p.; Bayer). Wound margins were infiltrated with local anesthetic (0.5% w/v bupivacaine; AstraZeneca). Animals were then placed in a stereotaxic frame (Kopf). Body temperature was maintained at 37 ± 0.5°C by a homeothermic heating device (Harvard Apparatus). Electrocorticograms (ECoGs) and respiration rate were monitored constantly to ensure the animals' well-being. The epidural ECoG was recorded with a 1-mm-diameter screw above the frontal (somatic sensory motor) cortex (4.2 mm rostral and 2.0 mm lateral of Bregma; Paxinos and Watson, 2007) and was referenced against a screw implanted above the ipsilateral cerebellum (Mallet et al., 2012; Abdi et al., 2015). Raw ECoG data were bandpass filtered (0.3–1500 Hz, −3 dB limits) and amplified (2000×; DPA-2FS filter/amplifier, NPI Electronic Instruments) before acquisition. Extracellular recordings of single-unit activity, that is, the action potentials (“spikes”) fired by individual neurons in the striatum were made using standard-wall borosilicate glass electrodes (10–30 MΩ *in situ*; tip diameter, ∼1.2 μm) containing 0.5 m NaCl solution and neurobiotin (1.5% w/v; Vector Laboratories; RRID:AB_2313575). Electrodes were lowered into the brain under stereotaxic guidance and using a computer-controlled stepper motor (IVM-1000, Scientifica), which allowed electrode placements to be made with submicron precision. Electrode signals were amplified (10×) through the bridge circuitry of an Axoprobe-1A amplifier (Molecular Devices), AC coupled, amplified another 100×, and filtered at 300–5000 Hz (DPA-2FS filter/amplifier). The ECoG and single-unit activity were each sampled at 16.7 kHz using a Power1401 Analog–Digital converter and a PC running Spike2 acquisition and analysis software (Cambridge Electronic Design). As described previously (Mallet et al., 2008a, 2012; Sharott et al., 2012; Abdi et al., 2015), single-unit activity in striatum was recorded during cortical slow-wave activity (SWA), which is similar to activity observed during natural sleep, and/or during episodes of spontaneous “cortical activation,” which contain patterns of activity that are more analogous to those observed during the awake, behaving state (Steriade, 2000). It is important to note that the neuronal activity patterns present under this anesthetic regime may only be qualitatively similar to those present in the unanesthetized brain. Nevertheless, the urethane-anesthetized animal still serves as a useful model for assessing the impact of extremes of brain state on functional connectivity within and between the basal ganglia and cortex in dopamine-intact and parkinsonian animals (Magill et al., 2006; Mallet et al., 2008a,b; Sharott et al., 2012). Importantly, excessive beta oscillations arise (in a brain state-dependent manner) in the basal ganglia and motor cortex of 6-OHDA-lesioned rats under this anesthetic regimen (Mallet et al., 2008a,b; Moran et al., 2011). Cortical activation was occasionally elicited by pinching a hindpaw for a few seconds. Note that we did not analyze neuronal activity recorded concurrently with the delivery of these sensory stimuli. Because the analyzed activity was recorded at least several minutes after the cessation of the brief pinch stimulus, it was also considered to be spontaneous (Mallet et al., 2008a). The animals did not exhibit a marked change in respiration rate, and did not exhibit a hindpaw withdrawal reflex, in response to the pinch. Moreover, withdrawal reflexes were not present during episodes of prolonged cortical activation, thus indicating that anesthesia was adequate throughout recordings. Following electrophysiological recordings, single striatal neurons were juxtacellularly labeled with neurobiotin (Sharott et al., 2012; Doig et al., 2014; Garas et al., 2016). Briefly, positive current pulses (2–10 nA, 200 ms, 50% duty cycle) were applied until the single-unit activity became robustly entrained by the pulses. Single-unit entrainment resulted in just one neuron being labeled with neurobiotin. Two to six hours after labeling, animals were killed and transcardially perfused with 100 ml of 0.05 m PBS, pH 7.4, followed by 300 ml of 4% w/v paraformaldehyde (PFA) in 0.1 m phosphate buffer (PB), pH 7.4. Brains were left overnight in fixative at 4°C and then stored for 1–3 d in PBS at 4°C before sectioning.

##### Electrical stimulation of motor cortex.

We used focal electrical stimulation of the motor cortex to test for the presence of striatal projection neurons that were effectively “quiescent” (Mallet et al., 2005, 2006; Kita and Kita, 2011; Escande et al., 2016), defined here as neurons that did not spontaneously fire for hundreds of seconds (see below) or at least exhibited very low rates of spontaneous firing [<0.03 spikes per second (spk/s)], during recording epochs without cortical stimulation. Parallel, bipolar, tungsten stimulating electrodes (constructed from nylon-coated stainless steel wires; California Fine Wire), with tip diameters of ∼100 μm, a tip separation of ∼150 μm, and an impedance of ∼10 kΩ, were implanted into the motor cortex ipsilateral to 6-OHDA lesions in urethane-anesthetized rats (Sharott et al., 2012). The coordinates of the cortical stimulation sites (2.0–3.0 mm rostral and 2.6–2.8 mm lateral of Bregma, at a depth of 2.0 mm below the dura) correspond approximately to layers 5/6 of primary motor cortex (Paxinos and Watson, 2007). Paired electrical stimuli, which consisted of two square-wave current pulses (each of 0.3 ms duration and 800 μA amplitude, with a 100 ms interval between each pulse), were delivered at a frequency of 0.5 Hz using a constant-current isolator (A360D, World Precision Instruments) that was gated by digital outputs from the Power1401 converter. Previous work in anesthetized 6-OHDA-lesioned rats suggests that these stimulation parameters are highly effective at evoking spike firing in striatal projection neurons, including those that do not fire spontaneously (Mallet et al., 2006; Ballion et al., 2009). The paired electrical stimuli were delivered to motor cortex while slowly (0.1–0.5 μm/s) advancing the glass electrode through the ipsilateral dorsal striatum; upon encountering a single unit that responded to the cortical stimulation, the electrode movement was stopped. After delivery of 10–20 paired stimuli to qualitatively establish that single-unit responses were of short and consistent latencies (<20 ms to first spike), the stimulation was halted and unit activity was recorded for 300–450 s (the epoch from which the spontaneous firing rate was calculated). Cortical stimulation then resumed for at least 50 trials to ensure that the same striatal single unit was still proximate to the recording electrode; when this was verified, the single neurons responsive to cortical stimulation were juxtacellularly labeled with neurobiotin and then recovered and processed for identification (Sharott et al., 2012; Garas et al., 2016). In this way, we sampled quiescent neurons blinded to cell type and then used *post hoc* anatomical methods to verify whether the same neurons were dSPNs or iSPNs (see below).

##### *In vivo* electrophysiological recording of striatal activity with multielectrode arrays.

Simultaneous extracellular recordings of unit activity and LFPs were made from numerous sites in the dorsal striatum of urethane-anesthetized control and 6-OHDA-lesioned rats using a linear array with multiple, spatially-defined recording contacts (“silicon probe”; NeuroNexus), as previously described (Magill et al., 2006; Mallet et al., 2008b). The probe had 16 recording contacts arranged in a single vertical plane, with a contact separation of 100 μm. Each contact had an impedance of 0.9–1.3 MΩ (measured at 1000 Hz) and an area of ∼400 μm^2^ (Magill et al., 2006). The probe was manually advanced into the dorsal striatum using a zero-drift micromanipulator (1760–1761; Kopf) under stereotaxic control (0.1–0.9 mm rostral and 2.6–3.4 mm lateral of Bregma; Paxinos and Watson, 2007) to final depths of 5.2–5.8 mm below the dura. The same probe was used throughout the series of experiments, but it was cleaned after each experiment in a proteolytic enzyme solution (Magill et al., 2006). This was sufficient to ensure that contact impedances and recording performance were not altered by probe use and reuse. Monopolar probe signals were recorded using high-impedance unity-gain operational amplifiers (Advanced LinCMOS, Texas Instruments) and were referenced against a screw implanted above the contralateral cerebellum. After initial amplification, extracellular signals were further amplified (1000×) and low-pass filtered at 6000 Hz using programmable differential amplifiers (Lynx-8, NeuraLynx). The ECoG and probe signals were each sampled at 16.7 kHz using a Power1401 converter and a PC running Spike2 software. After the recording sessions, animals were killed and transcardially perfused with fixative as described above. All recording locations were then verified using standard histological procedures (Magill et al., 2006).

##### Molecular characterization of recorded and juxtacellularly-labeled neurons.

Parasagittal sections (50 μm) were cut from each brain using a vibrating microtome (VT1000S, Leica), collected in series, and washed in PBS. Free-floating sections were then incubated overnight at room temperature in Triton PBS (PBS with 0.3% v/v Triton X-100 and 0.02% w/v sodium azide; Sigma-Aldrich) containing Cy3-conjugated streptavidin (1:3000 dilution; catalog #438315, Thermo Fisher Scientific). Sections containing neurobiotin-labeled neuronal somata and dendrites (those marked with Cy3) were then isolated for further examination. Neurobiotin-labeled neurons with densely spiny secondary and higher-order dendrites were classified as SPNs (Sharott et al., 2012; Garas et al., 2016). The few well labeled neurons that had aspiny dendrites (i.e., presumed interneurons; Sharott et al., 2012) were excluded from further analysis. Confirmed SPNs were additionally tested for the expression of preproenkephalin (PPE) by indirect immunofluorescence; somatic expression of PPE immunoreactivity was used to identify SPNs of the indirect pathway, whereas those SPNs that did not express PPE were considered to be direct pathway SPNs (Lee et al., 1997; Garas et al., 2016). To optimize immunolabeling for PPE in identified SPNs, we used a heat pretreatment as a means of antigen retrieval (Mallet et al., 2012; Abdi et al., 2015). After heat pretreatment, the sections were incubated for 1–2 h at room temperature in PBS containing 10% v/v normal donkey serum (NDS; Jackson ImmunoResearch, RRID:AB_2337258), and then incubated overnight at room temperature in PBS containing 1% v/v NDS and rabbit anti-PPE (1:5000; LS-C23084, Lifespan; RRID:AB_902714; Abdi et al., 2015; Garas et al., 2016). In some cases, the localization of SPNs with respect to striosomes/patches with enriched immunoreactivity for μ-opioid receptors (MOR1; Crittenden and Graybiel, 2011) was tested by simultaneously incubating sections in goat anti-MOR1 (1:300; catalog #sc-7488, Santa Cruz Biotechnology; RRID:AB_2156522). After exposure to primary antibodies, sections were washed in PBS and incubated overnight at room temperature in PBS containing secondary antibodies (all raised in donkey) that were conjugated to either Alexa Fluor 488 (1:500; Thermo Fisher Scientific; RRID:AB_141708) or DyLight 647 (1:500; Jackson ImmunoResearch; RRID:AB_2340437). All secondary antibodies were highly cross-adsorbed by the manufacturers to reduce cross-species reactivity. After washing in PBS, sections were mounted in Vectashield (Vector Laboratories) and imaged on an epifluorescence microscope (AxioImager.M2, Zeiss) and/or on confocal microscopes (LSM 510 or LSM 710, Zeiss) using the filters, laser settings, and protocols that we have previously detailed (Abdi et al., 2015). Images of each of the channels were taken sequentially and separately to negate possible cross talk of signal across channels. For a given molecular marker, X, we designate positive immunoreactivity (confirmed expression) as X^+^, and undetectable immunoreactivity (no expression) as X^−^. A juxtacellularly labeled SPN was classified as not expressing PPE only when PPE^+^ cells could be observed on the same optical section as the tested neuron.

##### Quantification of molecular marker expression in striatal projection neurons.

Four adult rats (age, 3–4 months; weight, 290–380 g) were killed with pentobarbital (1.5 g/kg, i.p.; Ayrton Saunders) and transcardially perfused with PBS followed by 4% w/v PFA in PB. Brains were left overnight in fixative at 4°C and then stored in PBS for 1–3 d at 4°C before being cut into 50-μm-thick coronal sections on a vibrating microtome. Sections of dorsal striatum that matched those targeted for electrophysiological recordings (i.e., from ∼1.5 mm rostral of Bregma to ∼0.5 mm caudal of Bregma; Paxinos and Watson, 2007) were then selected and processed for indirect immunofluorescence to reveal Ctip2 (also known as Bcl11b), a marker of all SPNs (Arlotta et al., 2008), PPE, and preprotachykinin A (PPTA), a precursor of the neuropeptide substance P that is selectively expressed by dSPNs (Lee et al., 1997). To optimize immunolabeling for PPE and PPTA in the somata of SPNs, we again used a heat pretreatment as a means of antigen retrieval (as above). After heat pretreatment, the sections were incubated for 1–2 h at room temperature in Triton PBS containing 10% v/v NDS and then incubated overnight at room temperature in Triton PBS containing 1% v/v NDS as well as rat anti-Ctip2 (1:500; catalog #ab18465, Abcam; RRID:AB_2064130; Garas et al., 2016), rabbit anti-PPE (as above), and guinea pig anti-PPTA (1:100; gift from T. Kaneko, Department of Morphological Brain Science, Graduate School of Medicine, Kyoto University, Japan; Lee et al., 1997). After exposure to primary antibodies, sections were washed in PBS and incubated overnight at room temperature in PBS containing secondary antibodies (all raised in donkey) that were conjugated to Alexa Fluor 488 (1:500; Thermo Fisher Scientific; RRID:AB_141709), DyLight 649 (1:500; Jackson ImmunoResearch; RRID:AB_2315775), or Cy3 (1:500; Jackson ImmunoResearch; RRID:AB_2340460). All secondary antibodies were highly cross-adsorbed by the manufacturers to reduce cross-species reactivity. After washing in PBS, sections were mounted in Vectashield (Vector Laboratories), and the dorsal striatum was imaged on an epifluorescence microscope (AxioImager.M2, Zeiss) running Axiovision software (Carl Zeiss) and equipped with a StereoInvestigator system (MBF Bioscience). Filter cubes were as previously detailed (Abdi et al., 2015). Images of each of the channels were taken sequentially and separately to negate possible cross talk of signal across channels.

Striatal projection neurons, defined by the expression of Ctip2 in their nuclei, were tested for the combinatorial expression of PPE and/or PPTA in their somatic cytoplasm. We used a version of design-based stereology, the “optical fractionator” (West, 1999, 2012) to generate unbiased cell counts and determine the proportions of Ctip2^+^ SPNs that were PPE^+^, PPTA^+^, PPE^+^/PPTA^+^, or PPE^−^/PPTA^−^. Briefly, ROIs (i.e., the borders of dorsal striatum) were first defined in sections using a 5× 0.16 numerical aperture (NA) objective lens. A series of tessellated, *z*-stacked images were then acquired using a 40× 1.3 NA oil-immersion objective lens and 1.0 μm steps (optical sections) at depths of 2–12 μm from the upper surface of each section. To minimize confounds arising from surface irregularities, neuropil within a 2-μm-thick “guard zone” at the upper surface was not imaged. This sampling strategy thus defined a 10-μm-thick “optical disector” that was used with unbiased 2D counting frames (120 × 85 μm; consisting of two perpendicular exclusion lines and two inclusion lines) to generate all cell counts and marker expression profiles (West, 1999, 2012; Glaser et al., 2007). Stereological sampling of dorsal striatum was randomized in each section tested. A given SPN was counted only once through the series of optical sections when its nucleus came into sharp focus within the disector (Abdi et al., 2015). A given SPN was classified as not expressing PPE or PPTA only when positive immunoreactivity for the respective marker could be observed in other SPNs on the same optical section as the tested neuron. The use of stereology, and this optical disector probe in particular, ensured that we could generate robust and unbiased cell counts in a timely manner. On average, 627 ± 32 (mean ± SEM) Ctip2^+^ SPNs were counted in each rat.

##### Analysis of basic firing parameters.

Data from the recording sessions were visually inspected, and epochs of robust cortical SWA or cortical activation were selected according to the previously described characteristics of these brain states (Mallet et al., 2006, 2008a,b; Sharott et al., 2012). A portion of the spike train recorded during each defined brain state was isolated and used for statistical analyses (average epoch durations of 460 ± 15 and 596 ± 13.4 s for recordings made with glass electrodes and silicon probes, respectively). Spike trains were assumed to be realizations of stationary stochastic point processes. Putative single-unit activity was isolated with standard “spike-sorting” procedures (Mallet et al., 2008a,b), including template matching, principal component analysis, and supervised clustering (Spike2). Isolation of a single unit was verified by the presence of a distinct refractory period in the interspike interval (ISI) histogram. For further analysis, single-unit activity was converted so that each spike was represented by a single digital event (Spike2). The mean firing rate (in spikes per second) was calculated from the total number of spikes per data epoch.

##### Analysis of phase-locked firing and circular statistics.

To investigate how the activity of individual striatal neurons varied in time with respect to ongoing cortical network activity, we analyzed the instantaneous phase relationships between striatal spike times and cortical oscillations in specific frequency bands (Sharott et al., 2012; Nakamura et al., 2014; Garas et al., 2016). Signal analyses were performed using MATLAB (MathWorks). Electrocorticogram signals containing robust SWA or cortical activation were initially band-pass filtered to isolate slow (0.4–1.6 Hz) or beta (15–30 Hz) oscillations, respectively (first- and second-order Butterworth filters for slow and beta oscillations). Subsequently, the instantaneous phase and power of the ECoG in these frequency bands were separately calculated from the analytic signal obtained via the Hilbert transform (Lachaux et al., 1999). In this formalism, peaks in the ECoG oscillations correspond to a phase of 0°, and troughs to a phase of 180°. Linear-phase histograms, circular-phase plots, and circular statistical measures were calculated using the instantaneous phase values for each spike. Descriptive and inferential circular statistics were then calculated using the CircStat toolbox (Berens, 2009) for MATLAB. For the calculation of vector lengths and statistical comparisons, we included only those neurons that fired ≥40 spikes during the entire analyzed epoch. These neurons were then tested for significantly phase-locked firing (defined as having *p* < 0.05 in Rayleigh's uniformity test). The null hypothesis for the Rayleigh's test was that the spike data were distributed in a uniform manner across/throughout the phase. We and others have previously remarked that the nonsinusoidal nature of some field potential oscillations, such as the cortical slow oscillation, can confound standard circular statistics, especially Rayleigh's test (Siapas et al., 2005; Mallet et al., 2008a,b; Sharott et al., 2012; Nakamura et al., 2014). Thus, for the analysis of striatal neuron firing relationships with cortical slow oscillations, Rayleigh's tests were performed only after any phase nonuniformities of the slow oscillations were corrected with the empirical cumulative distribution function (Siapas et al., 2005; Nakamura et al., 2014; Abdi et al., 2015; Garas et al., 2016). For each of the neurons that were significantly phase locked using these criteria, the mean phase angle was calculated. Differences in the mean phase angles of groups of neurons were tested for using the Watson–Williams *F* test (*p* < 0.05 for significance). The mean resultant vector length (referred to hereafter as simply “vector length”) of the distribution of instantaneous phase values for each spike, bound between 0 and 1 (the closer to 1, the more concentrated the angles), was used to quantify the level of phase locking around the mean phase for individual neurons (computed using the angles of each spike) and for populations of neurons (computed using the mean phase for each neuron). Where data are displayed in circular plots, lines radiating from the center are the vectors of the preferred phases of firing (with the center and perimeter of the outer grid circle representing vector lengths of 0 and 1, respectively); thin lines indicate preferred firing of individual neurons, whereas thick black lines indicate population vectors. The small open circles on the perimeter represent the preferred phases of each neuron.

##### Spectral analysis.

ECoGs and LFPs were low-pass filtered at 250 Hz and then downsampled to 500 Hz (MATLAB function “resample”). Spectral parameters for both time series were evaluated using fast Fourier transform (FFT), as described previously (Halliday et al., 1995), and power spectra were calculated with an FFT size of 2000 giving a frequency resolution of 0.25 Hz. The overlap of FFT windows was 50%. For analysis of LFPs recorded during cortical activation, all individual signals were rereferenced by subtracting the mean signal across all probe contacts to reduce volume conduction from nonstriatal sources. We also analyzed the “background-unit activities” (BUAs) recorded with silicon probes, a representation of the summed firing of small, local neuronal populations that is conceptually distinct from multiunit activity and LFPs (Moran and Bar-Gad, 2010). These BUAs were isolated from the wideband signals recorded with silicon probes by high-pass filtering off-line at 300 Hz (Spike2, finite impulse response filter) and, if necessary, after removing any large-amplitude action potentials that could potentially distort the signals and bias analyses (Moran et al., 2008; Moran and Bar-Gad, 2010). Large-amplitude action potentials were defined as those exceeding 4 SDs of the mean amplitude of the entire high-pass filtered signal and were removed (data points from 1 ms before to 3 ms after the action potential peak) and replaced with another randomly selected segment of the signal (of the same duration, and that did not contain similarly large action potentials). Background-unit activities were then low-pass filtered at 300 Hz (third-order Butterworth filter; MATLAB), downsampled to 2048 Hz, and rectified, so that they could be used as a continuous (time series) measure of the spiking activity of many neurons around the recording contact. A similar approach has been used for the isolation and analysis of BUAs recorded in the basal ganglia (Moran et al., 2008; Moran and Bar-Gad, 2010). The frequency resolution of spectra for BUAs recorded during SWA and cortical activation were 0.25 and 1 Hz, respectively. For some analyses of LFPs, each individual power spectrum was normalized to “% total power.” This was achieved by calculating the spectral power in each frequency bin as a percentage of the total power between 1 and 80 Hz (excluding the 49–51 Hz range that contained mains electrical noise in some recordings). Coherence spectra, used to assess the linear phase/amplitude relationships between time series, were calculated using the MATLAB toolbox Neurospec (version 2.0) for multivariate Fourier analyses (www.neurospec.org). Significance was evaluated using 95% confidence limits, based on the number of segments used, and were independent of frequency (Halliday et al., 1995). The square root of the coherence was Fisher transformed to normalize the variance before any averaging or statistical analysis (Halliday et al., 1995). Significance histograms were constructed by calculating the percentage of individual spectra where the value in a given frequency bin was greater than this confidence limit. For statistical comparison, the power or coherence averaged across all frequency bins in the band of interest was calculated, giving a single value for each recording.

##### Cross-correlation analysis.

Raw cross-correlograms (CCs) were calculated (5 ms bins with ±1 s lag) for every pair of single units recorded in striatum on different contacts of the silicon probe using a standard cross-correlation function (MATLAB function “xcorr”). For all CC-based analysis, a given pair of units was included in the group analysis only if the firing rates of both units were >;0.1 spk/s. In a first analysis, we aimed to detect pairs of spike trains where the number of coincidences and/or oscillatory properties were significantly different from those that would be predicted by their primary statistics (i.e., firing rate and ISI distribution). To this end, cross-correlations were calculated using surrogate spike trains constructed by globally shuffling the ISIs of both neurons in a given pair and calculating their cross-correlation 100 times (Sharott et al., 2009). This produced a null hypothesis distribution for each lag point. The raw correlation was then converted to a *z*-score (the number of SDs of the true correlation from the mean of the null hypothesis) that was used as a measure of the correlation strength because it is dependent mainly on the temporal locking of the two spike trains. A cross-correlation was considered significant at a given lag if it was outside two SDs of the null hypothesis; this criterion was used to construct significance CCs that were used to investigate the likelihood of a significant correlation between a specific pair type at a given lag. The use of these ISI-shuffled surrogates in this first analysis thus controlled for any differences in the firing rates and ISI distributions of spike train pairs from different ensembles of striatal units. In a second analysis, we aimed to detect pairs of spike trains where the number of coincidences and/or oscillatory properties were significantly different from those that would be predicted by their firing rate and the ways in which they phase locked their firing to cortical beta oscillations. To this end, cross-correlations were calculated and *z*-scored using surrogate spike trains in which spike times were reassigned based on the phases of the real spikes. These beta phase-shuffled surrogates had the same number of spikes as, and identical phase distributions to, the real data but were otherwise randomly placed in time. The *z*-score in this case tested against the null hypothesis that any features in the CC were the result of one or both units in a pair having a particular phase relationship with a third signal (the cortical beta oscillations). The oscillatory content of the correlation was evaluated by computing spectral parameters of the CCs (CC power). This conversion from time to frequency domain is advantageous for describing the oscillatory coupling of units with low firing rates (Sharott et al., 2009), where the spikes of one or both neurons may not oscillate, but still have a tendency to fire at specific time intervals (Sharott et al., 2009). To compute CC power, the power spectral density was calculated using the central 250 ms (i.e., from −125 to +125 ms) of the *z*-scored CC as nonoverlapping windows. Because the power was computed on the *z*-score, it therefore reflected oscillatory interaction that was not predicted by the primary spike train statistics or by the phase-locked firing of units. Because the CCs were often noisy, a multitaper power spectral density estimate (MATLAB function “pmtm”) was used to further smooth the spectral estimate. To measure the variance of this estimate for a given dataset (e.g., all putatively classified iSPN pairs), the power spectra density was calculated on 50% of the CCs selected at random. This procedure was repeated 1000 times to allow a mean and 99% confidence limits to be constructed for each group. Between groups, frequency bins at which these confidence limits did not overlap were considered to be significantly different.

##### Experimental design and statistical analyses.

For each experiment, descriptions of critical variables (e.g., number of animals, neurons, and other samples evaluated) as well as statistical design can be found in the Results. The Shapiro–Wilk test was used to judge whether noncircular datasets were normally distributed (*p* ≤ 0.05 to reject). Because some sets of continuous data were not normally distributed, we used nonparametric statistical testing for these throughout (MATLAB). The Mann–Whitney *U* test (MWUT) was used for comparisons of unpaired data. For multiple group comparisons, we performed a Kruskal–Wallis ANOVA on ranks, with Dunn's test for further *post hoc* definition of comparisons. For statistical exploration of whether dopamine depletion altered the proportions of spontaneously firing dSPNs and iSPNs *in vivo*, we used the Pearson's χ^2^ test (Excel, Microsoft) to assess the goodness of fit of the observed sample sizes of dSPNs and iSPNs, as electrophysiologically recorded in a manner blinded to cell type, to the expected sample sizes. The null hypothesis, which dictated the expected sample sizes, was that dSPNs and iSPNs would be recorded with equal incidence. Similarly, we used the Pearson's χ^2^ test to define whether dopamine depletion altered the proportions of neurons that phase locked their firing in time with ongoing cortical oscillations. The null hypothesis was that the incidence of phase-locked neurons in lesioned rats would be the same as that in controls. When the expected sample size was <10, we used the nonparametric binomial test (SPSS, IBM) instead of the χ^2^ test. Significance for all statistical tests was set at *p* < 0.05 (specific *p* values are given in the text). Data are represented as group means ± SEMs unless stated otherwise. All box plots in figures show the medians, the interquartile ranges (box), and extremes of the range (whiskers show the lowest and highest points within 1.5× the interquartile range, ∼99% of the data for a normal distribution).

## Results

The overall objective of this study was to define how the chronic depletion of dopamine, as occurs in PD, alters the temporal organization of electrical activity in the dorsal striatum *in vivo* at the level of single neurons, small neuronal ensembles and larger neuronal populations. Special emphasis was placed on defining whether and how the action potential firing of striatal neurons becomes entrained to the excessively synchronized beta-frequency oscillations that emerge in cortico-basal ganglia circuits after dopamine depletion. To address this, we sampled unit activities and LFPs from numerous sites in the striatum of anesthetized dopamine-intact rats and 6-OHDA-lesioned rats using linear arrays with multiple, spatially defined recording contacts (silicon probes). Neuronal activity dynamics in striatum were interrogated in the context of two well defined and controlled brain states, SWA and cortical activation, as verified in simultaneous recordings of electrocorticograms. To better resolve the potential contributions of different cell types to the striatal activity dynamics sampled with silicon probes, we also recorded the firing of individual identified spiny projection neurons of the direct pathway and indirect pathway under the same conditions.

### Dopamine depletion alters the rate, pattern, and synchronization of firing of striatal neurons during cortical slow-wave activity

**Figure 1.**
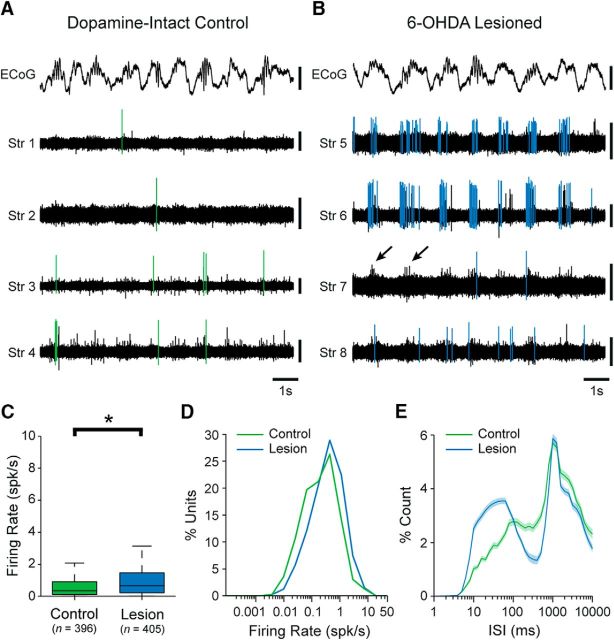
Unit activity in the dorsal striatum of dopamine-intact and 6-OHDA-lesioned rats during cortical slow-wave activity. ***A***, Striatal unit activity simultaneously recorded with a silicon probe during cortical slow-wave activity in a dopamine-intact control rat. Spikes fired by a single unit recorded on each of the striatal probe contacts (Str 1–4) are highlighted in green. During cortical slow-wave activity, the ECoG is dominated by a large-amplitude slow (∼1 Hz) oscillation. ***B***, Simultaneous recordings of striatal unit activity in a lesioned rat. Spikes fired by a single unit recorded on each of the striatal probe contacts (Str 5–8) are highlighted in blue. Note the rhythmic variations in the background-unit activity (arrows). ***C***, Mean firing rates of all striatal single units recorded in control and lesioned rats. On average, striatal units fired at significantly higher rates in lesioned rats. Number of single units included in each group is shown in parentheses. ***D***, Histogram of the firing rates of all single units in control and lesioned rats. ***E***, Normalized ISI histograms (mean ± SEM) of all single units in control or lesioned rats. Vertical calibration bars: ***A***, ***B***, 0.5 mV (ECoG); 0.1 mV (units). *p < 0.05 (Mann–Whitney *U* test).

Using silicon probes, we recorded the spontaneous action potential discharges (spikes) of 396 single units (neurons) in the dorsal striatum of dopamine-intact control rats (*n* = 8), and 405 striatal neurons in 6-OHDA-lesioned rats (*n* = 6), during cortical SWA (Fig. 1*A*,*B*). The majority of spontaneously active striatal neurons in control rats fired at low average rates (<1 spk/s) and with irregular patterns; neurons occasionally fired single spikes, or higher-frequency “bursts” of 2 or 3 spikes, around the peaks of the cortical slow (∼1 Hz) oscillations (Fig. 1*A*). Although many striatal neurons in lesioned rats fired in a manner similar to those in control rats, qualitative observations suggested an overall higher level of spontaneous activity in the striatum of lesioned rats, with some neurons faithfully firing bursts of spikes around the peaks of cortical slow oscillations (Fig. 1*B*). Accordingly, the mean firing rate of striatal neurons in lesioned animals (mean ± SEM, 1.20 ± 0.08 spk/s; range, 0.0057–16.54 spk/s) was significantly higher (MWUT, *p* = 4.50e-06; Fig. 1*C*) than that of neurons in control rats (mean, 0.79 ± 0.07 spk/s; range, 0.0044–14.76 spk/s). This small increase in the absolute firing rates of striatal neurons was equivalent to a substantial relative increase (∼50%) in their firing rates. It is reasonable to assume that the vast majority of the single units we recorded with silicon probes were SPNs, in part because the rodent striatum contains relatively small populations of interneurons (collectively, they likely constitute ∼5% of all striatal neurons). In line with this assumption, the low firing rates and irregular firing patterns of these units are similar to those of anatomically identified SPNs in anesthetized dopamine-intact and lesioned rats (Mallet et al., 2005, 2006; Sharott et al., 2012; Garas et al., 2016). As such, our recordings suggest that, during cortical SWA, chronic dopamine depletion is associated with significant increases in striatal “net output”.

**Figure 2.**
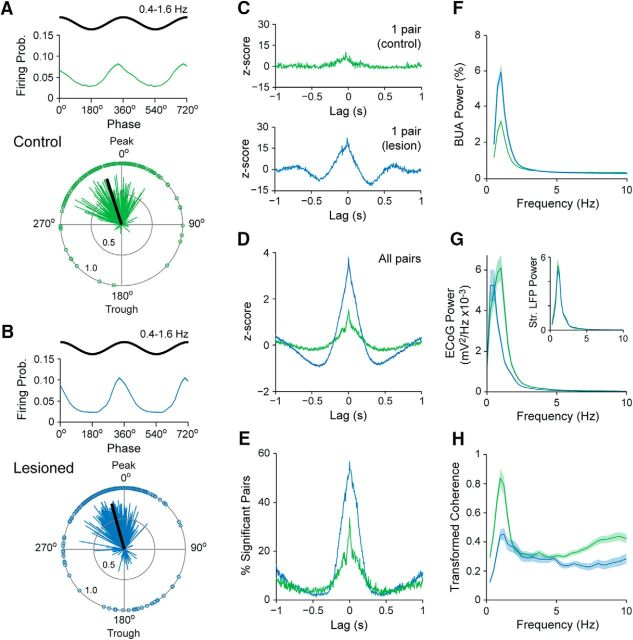
Temporal organization of single-unit and ensemble firing in the dorsal striatum of dopamine-intact and 6-OHDA-lesioned rats during cortical slow-wave activity. ***A***, ***B***, Mean linear-phase histograms of the firing of all striatal single units (top) and circular plots of the preferred firing angles of significantly phase-locked units (bottom), with respect to cortical slow oscillations (0.4–1.6 Hz) recorded in dopamine-intact control rats (***A***) and lesioned rats (***B***). In linear-phase histograms, two cycles of the cortical slow oscillation are shown for clarity. In circular plots, vectors of the preferred firing of individual units are shown as thin lines radiating from the center. Greater vector lengths indicate lower variance in the distribution of spikes around the mean phase angle of an individual unit. Each circle on the plot perimeter represents the preferred phase angle of an individual unit. Thick black lines radiating from the center indicate the mean phase angle of all striatal units in that group. Note that striatal units in control and lesioned rats tended to fire just before the peak (0°/360°) of the cortical slow oscillation. ***C***, Examples of normalized (*z*-scored) cross-correlograms for a pair of striatal single units recorded during cortical slow-wave activity in a control rat (green) and for another pair of single units recorded in a lesioned rat (blue). ***D***, Mean normalized cross-correlograms for all striatal unit pairs recorded in controls (green) and lesioned rats (blue). ***E***, Histograms of significant, positive correlations (*z*-score >; 2) in spike firing for all pairs of striatal units in controls (green) and for all pairs of units in lesioned rats (blue). Note that histograms of unit pairs in lesioned rats exhibited larger central peaks with clearer side lobes, indicating more highly synchronized firing with a more pervasive slow oscillatory component. ***F***, Mean power spectra of all measures of striatal BUA in controls (green) and lesioned rats (blue). ***G***, Mean power spectra of all ECoGs that were simultaneously recorded with striatal signals in controls (green) and lesioned rats (blue). Inset shows mean power spectra of the respective striatal LFPs (Str. LFP). ***H***, Mean transformed coherence between all ECoG–LFP pairs in controls (green) and lesioned rats (blue). Shaded areas in ***A***, ***B***, ***F–H*** show SEMs. Prob., Probability.

We next examined whether and how the spike firing of striatal neurons is temporally related to the stereotyped cortical slow oscillations prevalent during SWA. We thus used the Hilbert transform to analyze the instantaneous phase of the spiking of striatal neurons with respect to ECoG oscillations at 0.4–1.6 Hz (Sharott et al., 2012; Nakamura et al., 2014; Abdi et al., 2015). To qualify for these and related circular statistical analyses, a striatal neuron had to fire ≥40 spikes during the recording, a sampling criterion that helped to ensure accurate determination of circular means and the significance of any phase-locked firing. In both control and lesioned rats, qualifying striatal neurons tended to discharge just before the peak (0°/360°) of the cortical slow oscillation (Fig. 2*A*,*B*). This phase-locked firing is in good agreement with that previously reported for identified SPNs recorded during SWA in anesthetized rats (Mallet et al., 2005; Sharott et al., 2012; Garas et al., 2016). The mean angles of firing of significantly phase-locked neurons (defined using Rayleigh's uniformity test) in control and lesioned rats (342.1 ± 2.7°, *n* = 191 neurons, in controls; 344.5 ± 2.2°, *n* = 314 neurons, in lesioned) were similar, as were the population vector lengths for each group (control, 0.79; lesioned, 0.77; Fig. 2*A*,*B*). However, the proportion of striatal neurons that fired in a significantly phase-locked manner in lesioned rats (87% of neurons) was significantly higher (Pearson's χ^2^, *p* = 2.22e-24) than that in control rats (60.8%).

We also examined whether and to what extent pairs of striatal units fired in a temporally correlated manner during SWA (Fig. 2). Cross-correlograms of pairs of striatal neurons recorded from control rats often exhibited small and broad peaks that were centered around zero lag (Fig. 2*C*, top), which is in agreement with our observation that the firing of most neurons occurred near the peak of the cortical slow oscillation (Fig. 2*A*). The CCs for unit pairs in lesioned rats often exhibited larger central peaks with clearer “side lobes” (Fig. 2*C*, bottom), indicating a more pervasive slow oscillatory component in their synchronized firing. The *z*-scores of the CCs at zero lag were significantly greater in lesioned rats than in controls rats (*n* = 915 and 490 pairs, respectively; MWUT, *p* = 1.54e-31; Fig. 2*D*). In line with this, approximately half of all striatal unit pairs in the lesioned rats exhibited significant positive correlations at zero lag, whereas only a quarter of unit pairs in controls were correlated (Fig. 2*E*). To gain insight into whether and how these alterations in striatal activity extended to the collective outputs from larger ensembles of neurons, we next analyzed the background-unit activity signals (Fig. 1*B*) that represent the firing of many neurons around the probe contacts (Moran et al., 2008; Moran and Bar-Gad, 2010). When these BUA signals are used as a continuous time series, they enable spectral analyses that are relatively independent of firing rate. The spectral power of the BUA signals at slow oscillation frequencies (0.4–1.6 Hz) was considerably higher in lesioned rats than in controls (lesioned rats, *n* = 592 probe channels; controls, *n* = 864 probe channels; MWUT, *p* = 4.82e-39; Fig. 2*F*), again suggesting that large ensembles of striatal neurons are inappropriately recruited to the slow oscillations after dopamine depletion.

The analyses above show that striatal neurons in 6-OHDA-lesioned rats had higher firing rates, higher incidences of phase-locked firing to cortical slow oscillations, and higher levels of synchronized firing. These alterations in striatal activity dynamics could arise from systematic increases in the low-frequency oscillatory activity of cortical neurons after dopamine depletion. However, this was unlikely because ECoG power in the frequency band incorporating the slow oscillation (0.4–1.6 Hz) was slightly lower in lesioned rats compared with controls (MWUT, *p* = 0.002; Fig. 2*G*), which is in agreement with previous studies (Mallet et al., 2006). Moreover, power spectra of striatal LFPs recorded in lesioned and control rats were similar (Fig. 2*G*). In line with the decreased power of cortical slow oscillations in lesioned rats, the coherence at slow oscillation frequencies between ECoGs and striatal LFPs in lesioned rats was about half of that in controls (lesioned rats, *n* = 36 ECoG–LFP pairs; controls, *n* = 54 ECoG–LFP pairs; MWUT, *p* = 2.21e-04; Fig. 2*H*). Overall, these results suggest that increases in synchronized, slow oscillatory output from striatum arises after dopamine depletion despite potential decreases in slow oscillatory output from the cortex.

In summary, these silicon probe recordings demonstrate that the firing of individual striatal neurons in dopamine-intact control rats and 6-OHDA-lesioned rats was phase locked to the cortical slow oscillation with similar timing and precision. However, chronic dopamine depletion was associated with increases in the firing rates of a subpopulation of striatal neurons and an increase in the low-frequency oscillatory, synchronized output of neuronal ensembles.

### Dopamine depletion increases the firing rates of identified spiny projection neurons during cortical slow-wave activity

**Figure 3.**
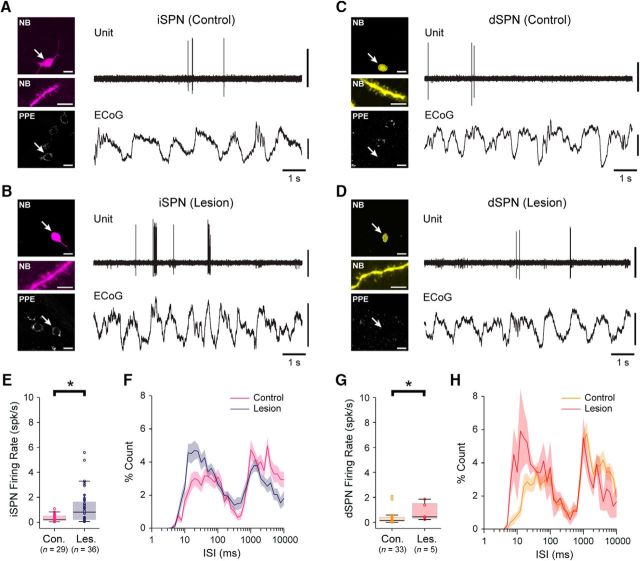
Spontaneous firing of indirect pathway SPNs and direct pathway SPNs during cortical slow-wave activity in dopamine-intact and 6-OHDA-lesioned rats. ***A***, ***B***, Left side, single-plane confocal fluorescence micrographs of indirect pathway SPNs, identified after labeling with neurobiotin (NB) by their densely spiny dendrites (middle panels), in a dopamine-intact control rat (***A***) and a lesioned rat (***B***). Both SPNs (arrows) expressed immunoreactivity for PPE, confirming them to be iSPNs (bottom). Also see Fig. 3-1. Right side, The action potentials spontaneously fired by the same identified iSPNs (unit) during cortical slow-wave activity, as verified in ECoG recordings. Note that, after dopamine depletion, iSPNs tend to fire spikes more frequently. ***C***, ***D***, Micrographs of NB-labeled direct pathway SPNs in a control rat (***C***) and a lesioned rat (***D***). Neither SPN expressed immunoreactivity for PPE, identifying them as dSPNs. ***E***, Firing rates of identified iSPNs in control (Con.) and lesioned (Les.) rats. On average, iSPNs fired at significantly higher firing rates in lesioned rats. Number of SPNs included in each group is shown in parenthesis. ***F***, Mean ISI histograms for iSPNs recorded in control or lesioned rats (shaded areas show SEMs). ***G***, Firing rates of identified dSPNs in control and lesioned rats. On average, dSPNs fired at significantly higher firing rates in lesioned rats. ***H***, Mean ISI histograms for dSPNs. Scale bars: ***A–D***, 20 μm; images of dendrites, 5 μm. Vertical calibration bars: ***A–D***, 0.5 mV (ECoG); 1 mV (units). *p < 0.05 (Mann–Whitney *U* test).

The direct/indirect pathways model of cortico-basal ganglia circuit organization posits that the loss of dopamine from these circuits results in an imbalance in the two striatal output pathways, such that the activities of iSPNs and dSPNs are inappropriately increased and decreased, respectively (DeLong, 1990; Smith et al., 1998). It thus follows that these two cell types might make different contributions to the overall or net changes in striatal activity dynamics that we observed in our silicon probe recordings. To address this possibility and to gain more insight into the firing of specific cell types *in vivo*, we used a single-cell recording/labeling method that allows for the direct and unambiguous correlation of the spike firing of an individual neuron with its structural and/or molecular properties (Sharott et al., 2012; Garas et al., 2016). Thus, using glass electrodes containing the tracer neurobiotin, we first recorded individual striatal neurons in control and lesioned rats (*n* = 37 and 15 rats, respectively) during cortical SWA (Fig. 3). We then juxtacellularly labeled each recorded neuron with neurobiotin for *post hoc* verification of their location and structural properties; neurobiotin-labeled striatal neurons giving rise to densely spiny dendrites were identified as SPNs (Sharott et al., 2012; Garas et al., 2016). Each recorded and identified SPN was additionally tested for somatic expression of immunoreactivity for PPE, a precursor of the neuropeptide enkephalin that is selectively expressed by SPNs of the indirect pathway (Lee et al., 1997; Gerfen and Surmeier, 2011; Fig. 3). To verify the utility and reliability of the somatic expression of PPE immunoreactivity as a selective marker of iSPNs in rat dorsal striatum, we performed stereological analyses of immunofluorescence signals for Ctip2, a transcription factor expressed in all SPNs but not in other major cell types in striatum (Arlotta et al., 2008), PPE, and PPTA, a precursor of the neuropeptide substance P that is selectively expressed by SPNs of the direct pathway (Lee et al., 1997). We used these data to generate unbiased estimates of the proportions of SPNs that express PPE, PPTA, both PPE and PPTA, or neither marker. Qualitative observations suggested that the vast majority of SPNs expressed either PPE or PPTA, such that the coexpression or absence of both of these markers in SPNs was rare (Fig. 3-1). Our cell counts revealed that, on average, 48.2 ± 1.8% of SPNs expressed PPE (but not PPTA), 47.6 ± 1.6% of SPNs expressed PPTA (but not PPE), 1.7 ± 0.2% of SPNs coexpressed PPE and PPTA, and 2.5 ± 0.9% of SPNs expressed neither PPE or PPTA (*n* = 2509 Ctip2^+^ SPNs counted in 4 rats; Fig. 3-1). These counts indicate that >;96% of SPNs expressing PPE (PPE^+^) are iSPNs, whereas >;94% of SPNs that do not express PPE (PPE^−^) are dSPNs. Together, these data confirm that, in rat dorsal striatum, the somatic expression of PPE immunoreactivity is a highly reliable and selective marker for iSPNs (Lee et al., 1997); the absence of somatic PPE immunoreactivity in SPNs is a similarly valid marker of dSPNs. For the purposes of classifying the SPNs that we recorded and neurobiotin labeled *in vivo*, all PPE^+^ SPNs were considered to be iSPNs, whereas all PPE^−^ SPNs were considered to be dSPNs (Garas et al., 2016).

10.1523/JNEUROSCI.0658-17.2017.f3-1 Figure 3-1 Somatic expression of preproenkephalin immunoreactivity is a highly reliable marker for distinguishing between spiny projection neurons of the indirect and direct pathways in rat dorsal striatum. *A*, Immunofluorescence signal for preproenkephalin (PPE), a selective marker of iSPNs. *B*, Signal for preprotachykinin A (PPTA), a selective marker of dSPNs. *C*, Signal for Ctip2, a transcription factor expressed by all SPNs. *D*, Merge of signals for PPE, PPTA and Ctip2. Immunoreactivities for PPE and PPTA were localized to the somatic cytoplasm of SPNs, whereas Ctip2 was localized to the nuclei of SPNs. Note that, in general, PPE-expressing SPNs (arrowheads) do not co-express PPTA. Similarly, most PPTA-expressing SPNs (arrows) do not co-express PPE. *E*, Proportions of all SPNs, defined with Ctip2, expressing the given molecular markers. Gray bars represent grand averages from all rats tested, and each black dot represents the average from an individual rat. Note that the vast majority of SPNs in rat dorsal striatum express either PPE or PPTA, and that PPE+/PPTA− SPNs and PPTA+/PPE− SPNs are equally abundant. Scale bar: *D*, 25 µm (also applies to *A*-*C*). Download Figure 3-1, TIF file

We recorded and juxtacellularly labeled 62 SPNs in dopamine-intact control rats and 41 SPNs in 6-OHDA-lesioned rats during SWA (Fig. 3). In good agreement with the data and interpretations arising from our silicon probe recordings, many of the spontaneously active SPNs fired at low average rates (<1 spk/s) and with irregular patterns; neurons sporadically fired single spikes, or higher-frequency bursts of 2 or 3 spikes, around the peaks of the cortical slow oscillations (Fig. 3*A–D*). This held true for many PPE^+^ iSPNs and PPE^−^ dSPNs, regardless of whether they were recorded in control or lesioned rats (Fig. 3*A–D*). However, and also in accordance with our silicon probe data, the average firing rate of all SPNs recorded in lesioned rats (1.15 ± 0.20 spk/s) was significantly higher (MWUT, *p* = 0.00003) than that of SPNs recorded in controls (0.32 ± 0.05 spk/s). Of the SPNs recorded in control rats, 29 were identified as iSPNs (Fig. 3*A*) and 33 were identified as dSPNs (Fig. 3*C*). Of the SPNs recorded in lesioned rats, 36 were identified as iSPNs (Fig. 3*B*) and 5 were identified as dSPNs (Fig. 3*D*). Qualitative observations suggested that the activity of many iSPNs (Fig. 3*B*), but rarely of dSPNs (Fig. 3*D*), was markedly increased in lesioned rats. Accordingly, the average firing rate of iSPNs in lesioned rats (1.20 ± 0.04 spk/s) was significantly higher (MWUT, *p* = 0.003; Fig. 3*E*) than that of iSPNs in control rats (0.34 ± 0.01 spk/s). This increase in the absolute firing rates of iSPNs was equivalent to a substantial relative increase (∼250%) in their firing rates. The average firing rate of dSPNs in lesioned rats (0.84 ± 0.15 spk/s) was also significantly higher (MWUT, *p* = 0.034; Fig. 3*G*) than that of dSPNs in control rats (0.31 ± 0.01 spk/s). This increase in the absolute firing rates of dSPNs was equivalent to a ∼170% increase in their relative firing rates, which is surprising given previous electrophysiological studies reporting that the activity of dSPNs is strongly depressed after 6-OHDA lesions (Mallet et al., 2006). However, it should be noted that, although approximately equal numbers of iSPNs and dSPNs were recorded (blinded to cell type) in control animals, our sample of iSPNs in lesioned rats was approximately seven times larger than our sample of dSPNs in lesioned rats. Our stereological analyses indicated that iSPNs and dSPNs are equally abundant in the areas of dorsal striatum that we targeted for electrophysiological recordings. Thus, if the proportion of all iSPNs that were spontaneously firing (meaning they could be registered by our extracellular recordings) was similar to the proportion of all dSPNs that were firing, then each cell type should be sampled with the same incidence during recordings. The actual sample sizes of iSPNs and dSPNs recorded in control rats were not different from those expected from equal sampling of similarly active populations (Pearson's χ^2^, *p* = 0.61). However, the sample sizes of iSPNs and dSPNs recorded in lesioned rats were significantly different from those expected (Pearson's χ^2^, *p* = 1.29e-06). Thus, as previously suggested (Mallet et al., 2006; Ballion et al., 2009), it is likely that, after chronic dopamine depletion, a greater proportion of dSPNs are silent during cortical SWA.

**Figure 4.**
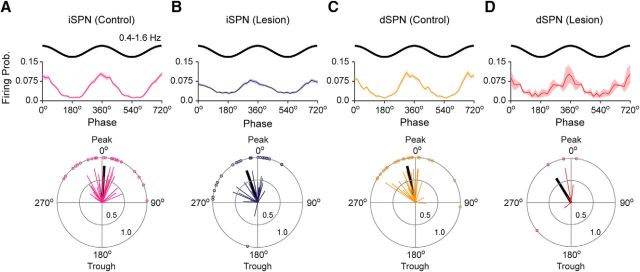
Firing of indirect pathway SPNs and direct pathway SPNs with respect to cortical slow oscillations in dopamine-intact rats and 6-OHDA-lesioned rats. ***A***, ***B***, Mean linear phase histograms of the firing of all identified iSPNs (top) and circular plots of the preferred firing angles of significantly phase-locked iSPNs (bottom) recorded in dopamine-intact control rats (***A***) and lesioned rats (***B***). For clarity, two cycles of the cortical slow oscillation (0.4–1.6 Hz) are shown in linear-phase histograms (shaded areas show SEMs). Thick black line in each circular plot indicates the mean phase angle of that group of SPNs. In both controls and lesioned rats, iSPNs tended to phase lock their firing around the peaks of the cortical slow oscillations. ***C***, ***D***, Mean linear-phase histograms and circular plots of the firing of identified dSPNs recorded in control rats (***C***) and lesioned rats (***D***). The dSPNs also tended to fire around the peaks of the cortical slow oscillations. Prob., Probability.

We next defined how the spike firing of iSPNs and dSPNs is temporally related to the cortical slow oscillation (Fig. 4). The firing of >;75% of qualifying iSPNs and dSPNs in control and lesioned rats was significantly phase locked to slow oscillations. In control rats, iSPNs tended to discharge at the peak of the cortical slow oscillation (0.9 ± 7.2°; *n* = 24 iSPNs; Fig. 4*A*), and dSPNs just before the peak of the slow oscillation (350.1 ± 7.4°; *n* = 20 dSPNs; Fig. 4*C*). The population vector lengths for iSPNs and dSPNs were similar (0.81 and 0.84, respectively; Fig. 4*A*,*C*). In lesioned rats, both iSPNs and dSPNs tended to discharge just before the peak of the cortical slow oscillation (Fig. 4*B*: 340.9 ± 8.3°; *n* = 24 iSPNs; Fig. 4*D*: 328.5 ± 24.2°; *n* = 4 dSPNs). Dopamine depletion did not result in significant changes to the mean angles of firing of iSPNs and dSPNs. However, the population vector lengths for iSPNs and dSPNs in lesioned rats (0.74 and 0.64, respectively; Fig. 4*B*,*D*) were reduced by 9% and 24% compared with those of SPNs in controls (Fig. 4*A*,*C*), thus suggesting less consistency in the phase-locked firing of dSPNs in particular after dopamine depletion.

In summary, these recordings of individual identified iSPNs and dSPNs demonstrate that, when the dopamine system is intact, these two cell types cannot be readily distinguished on the basis of their spontaneous firing rates/patterns during SWA *in vivo*. Dopamine depletion was associated with increases in the firing rates of both iSPNs and dSPNs, although the relative increase and upper range of firing rates were larger for iSPNs. Moreover, after dopamine depletion, spontaneously firing iSPNs were more prevalent than spontaneously firing dSPNs. With our silicon probe recordings in mind, it is most likely that iSPNs are the major contributors to the increases in overall firing rate and level of low-frequency oscillatory, synchronized firing that were observed in the dopamine-depleted striatal network during cortical SWA.

### Dopamine depletion alters the rate and beta-frequency synchronization of striatal neuron firing during cortical activation

**Figure 5.**
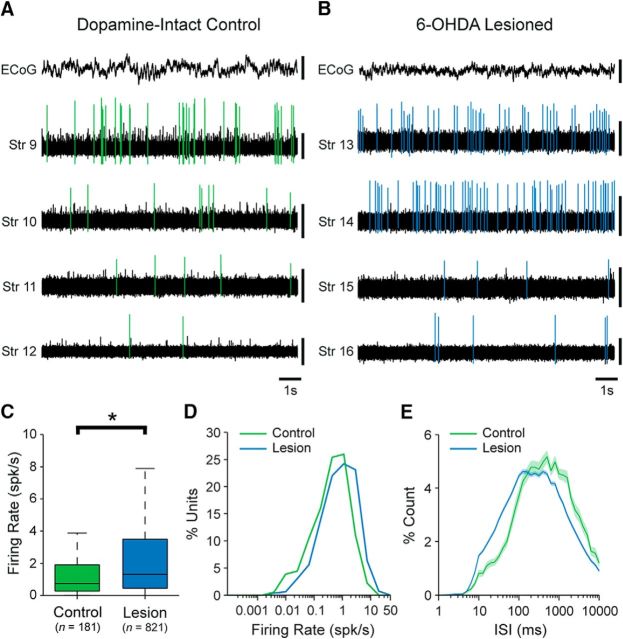
Unit activity in the dorsal striatum of dopamine-intact and 6-OHDA-lesioned rats during spontaneous cortical activation. ***A***, Striatal unit activity simultaneously recorded with a silicon probe during cortical activation in a dopamine-intact control rat. Spikes fired by a single unit recorded on each of the striatal probe contacts (Str 9–12) are highlighted in green. During the activated brain state, cortical activity is dominated by relatively small-amplitude high-frequency oscillations, as verified in ECoG recordings. ***B***, Simultaneous recordings of striatal unit activity in a lesioned rat. Spikes fired by a single unit recorded on each of the striatal probe contacts (Str 13–16) are highlighted in blue. ***C***, Mean firing rates of all striatal single units recorded in control and lesioned rats. On average, striatal units fired at significantly higher rates in lesioned rats. Number of single units included in each group is shown in parentheses. ***D***, Histogram of the firing rates of all single units in control and lesioned rats. ***E***, Normalized ISI histograms (mean ± SEM) of all single units in control or lesioned rats. Vertical calibration bars: ***A***, ***B***, 0.5 mV (ECoG); 0.1 mV (units). *p < 0.05 (Mann–Whitney *U* test).

Although exaggerated beta oscillations (15–30 Hz) have been recorded in striatal LFPs during the activated brain state in 6-OHDA-lesioned rats (Moran et al., 2011), it is not known whether and to what extent these rhythms are represented in the suprathreshold activity (spike firing) of striatal neurons. Defining the spike firing dynamics of striatal neurons is a prerequisite for understanding the roles they might play in the generation and/or dissemination of exaggerated beta oscillations. To address these issues, we used silicon probes to record the spontaneous activity of 181 single units in the dorsal striatum of dopamine-intact control rats (*n* = 6) and 821 striatal neurons in 6-OHDA-lesioned rats (*n* = 7) during cortical activation (Fig. 5*A*,*B*). Compared with SWA, cortical activation is exemplified by a large decrease in (a relative paucity of) cortical slow oscillations (Figs. 1, 5). Accordingly, cortical activation in control rats and lesioned rats was accompanied by reductions of 87% and 62%, respectively, in ECoG power at 0.4–1.6 Hz compared with that during SWA. These reductions across brain state were significant, but there was no difference in residual ECoG power at 0.4–1.6 Hz during activation in control and lesioned rats (Kruskal–Wallis ANOVA, *p* = 3.60e-27, χ^2^ = 117, with *post hoc* Dunn's tests). The majority of spontaneously active striatal neurons in control rats fired at low average rates (<2 spk/s) and with irregular patterns; neurons fired single spikes and/or brief bursts of spikes every few seconds (Fig. 5*A*). Although many striatal neurons in lesioned rats fired in a manner similar to those in control rats, qualitative observations revealed that many other neurons fired at high rates that were rarely seen in control rats (Fig. 5*B*). This was supported by quantitative analyses; the mean firing rate of striatal neurons in lesioned rats (2.62 ± 0.12 spk/s; range, 0.004–31.0 spk/s) was significantly higher (MWUT, *p* = 5.65e-07; Fig. 5*C*) than that of neurons in control rats (1.45 ± 0.16 spk/s; range, 0.004–17.63 spk/s). This small increase in the absolute firing rates of striatal neurons was equivalent to a substantial relative increase (∼80%) in their firing rates. A comparison of the firing rates of all striatal units recorded in control or lesioned rats during SWA or cortical activation (Figs. 1, 5) revealed a highly significant difference across all four groups of neurons (control SWA, lesioned SWA, control activated, lesioned activated; Kruskal–Wallis ANOVA, χ^2^ = 211, *p* = 1.60e-41). *Post hoc* testing (Dunn's tests) revealed that the average firing rate of striatal neurons in lesioned rats during activation was significantly higher than those in the other three groups; the average firing rate of striatal neurons in control rats was higher during activation than during SWA, and the average firing rate of striatal neurons in lesioned rats during SWA was higher than those of neurons in control rats during SWA. Together, these data demonstrate that not only is the average firing rate of striatal neurons increased during transitions in brain state from SWA to activation but also that dopamine depletion is associated with an overall increase in striatal neuron firing in the activated state.

**Figure 6.**
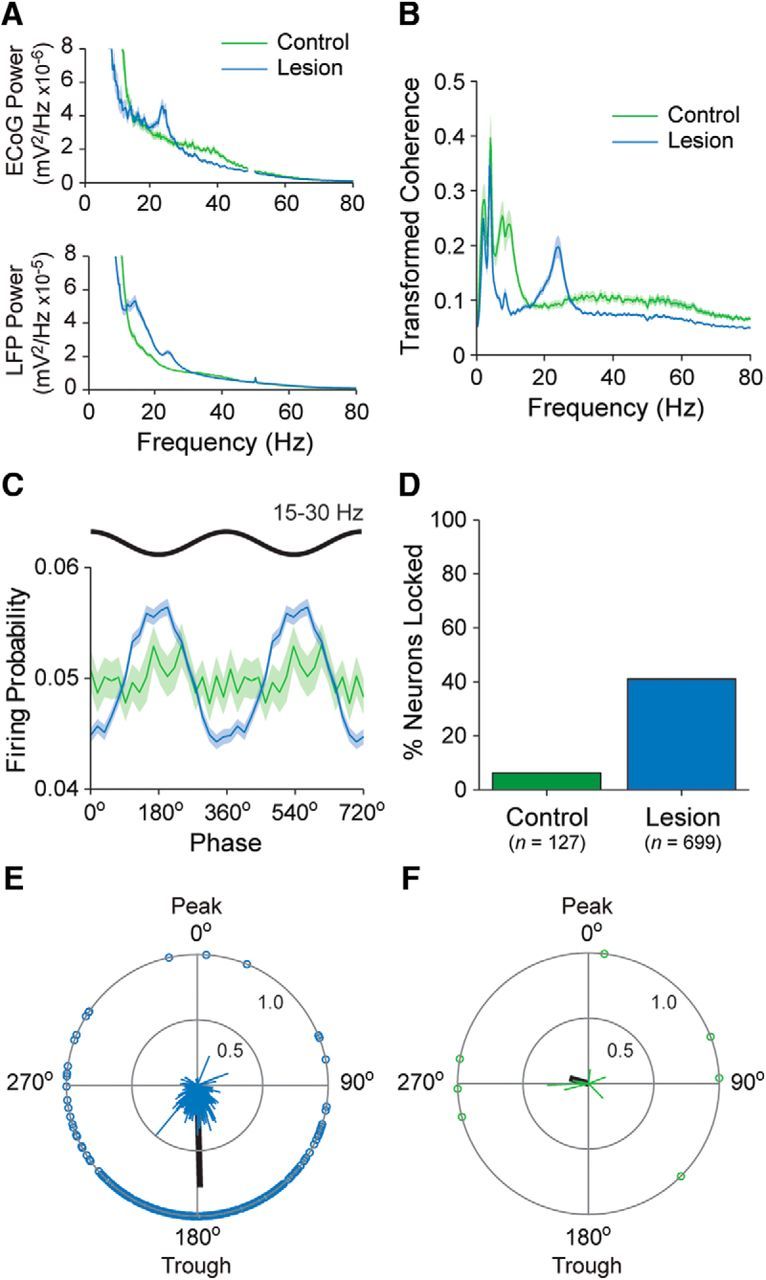
Temporal organization of striatal single-unit activity with respect to cortical beta oscillations in dopamine-intact rats and 6-OHDA-lesioned rats. ***A***, Mean power spectra of all ECoGs and all striatal LFPS simultaneously recorded during spontaneous cortical activation in dopamine-intact control rats (green) and lesioned rats (blue). ***B***, Mean transformed coherence between all ECoG–LFP pairs in controls and lesioned rats. Note the peak in coherence in the beta-frequency range (15–30 Hz) in lesioned rats. ***C***, Mean linear-phase histograms of the firing of all striatal single units with respect to the cortical beta oscillations (15–30 Hz) recorded during the activated brain state in controls (green) and lesioned rats (blue). For clarity, two cortical beta-oscillation cycles are shown. ***D***, Proportions of striatal single units that fired in a significantly phase-locked manner to cortical beta oscillations in controls and lesioned rats. Total numbers of striatal units tested are in parentheses. Note that, after dopamine depletion, a much larger proportion of striatal units fired in time with the cortical beta oscillations. ***E***, ***F***, Circular plots of the preferred firing angles of significantly phase-locked striatal units recorded in lesioned rats (***E***) and controls (***F***). Note that, in lesioned rats, striatal units tended to fire around the troughs of the cortical beta oscillations. Shaded areas in ***A–C*** show SEMs.

The power spectra of ECoGs recorded in lesioned rats during the activated brain state often exhibited discrete peaks in the beta-frequency range (15–30 Hz), as reported previously (Mallet et al., 2008a,b; Moran et al., 2011). The ECoG power in the center of this frequency range (20–25 Hz) was on average significantly higher in lesioned rats than in controls (MWUT, *p* = 0.02; Fig. 6*A*). There was also a broader peak at beta frequencies in the power spectra of the striatal LFPs simultaneously recorded in lesioned rats, and LFP power over the whole beta range was significantly greater in lesioned rats compared with controls (MWUT, *p* = 8.26e-10; Fig. 6*A*; *n* = 1170 and 560 LFP recordings, respectively). In line with these increases in beta oscillation power, there was marked coherence at beta frequencies (15–30 Hz) between ECoGs and striatal LFPs in lesioned rats (Fig. 6*B*), with beta coherence in lesioned rats being significantly higher (MWUT, *p* = 0.02) than that in controls (controls, *n* = 560 ECoG–LFP pairs; lesioned rats, 1440 ECoG–LFP pairs). We quantified the temporal relationship between the cortical beta oscillations and the spike firing of striatal neurons in lesioned and control rats. Striatal single units in lesioned rats (*n* = 699 neurons), but not those in control rats (*n* = 127 neurons), exhibited a clear tendency to discharge around the troughs of the cortical beta oscillations (Fig. 6*C*). In lesioned rats, 41% of striatal neurons fired in a significantly phase-locked manner to the beta oscillations, whereas in control rats, only 6% of neurons did so (Fig. 6*D*). The observed proportion of neurons with phase-locked firing in lesioned rats was significantly different (i.e., much larger) than the proportion expected from recordings in controls (Pearson's χ^2^, *p* < 0.0001e-100). The vast majority (94%) of the significantly phase-locked neurons in lesioned rats (*n* = 287 neurons) preferentially fired around beta oscillation troughs, defined as a phase angle of between >;90° and <270°. The mean angle of firing of these neurons in lesioned rats was 178.6 ± 2.2°, and the population vector length was relatively large (0.75), thus confirming a consistent preference to fire around the beta oscillation troughs (Fig. 6*E*). In contrast, the few striatal neurons in controls rats that had significantly phase-locked firing (*n* = 8 neurons) showed no clear or consistent preference for any phase angle of the cortical beta oscillations (Fig. 6*F*).

**Figure 7.**
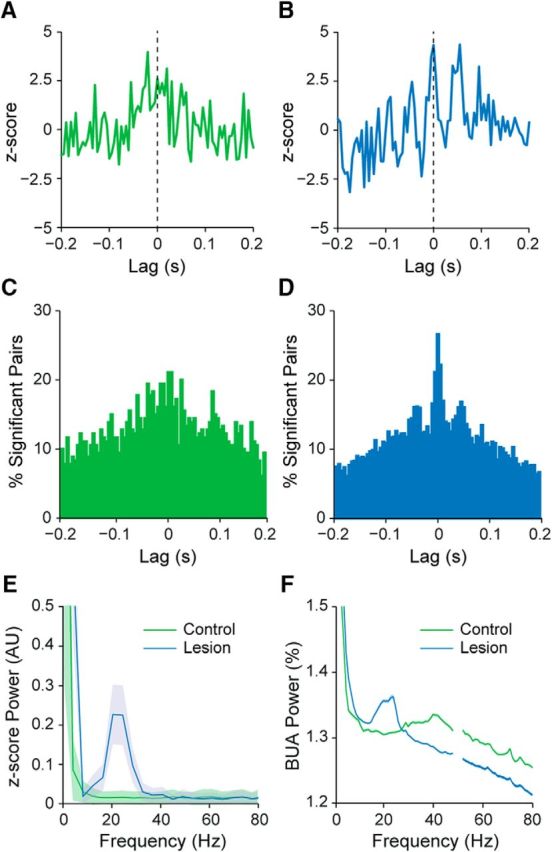
Synchronization of striatal unit activity during cortical activation in dopamine-intact and 6-OHDA-lesioned rats. ***A***, ***B***, Representative examples of normalized (*z*-scored) cross-correlograms for a pair of striatal single units recorded during cortical activation in a control rat (***A***, in green) and for another pair of single units recorded in a lesioned rat (***B***, in blue). ***C***, ***D***, Histograms of significant, positive correlations (*z*-score >; 2) in spike firing for all pairs of striatal units in controls (***C***, in green) and for all pairs of units in lesioned rats (***D***, in blue). Note that cross-correlations of unit pairs in lesioned rats often exhibited comparatively higher central peaks and more prominent side lobes with intervals of 40–50 ms, indicating a more prevalent beta oscillatory component in their synchronized firing. ***E***, Power spectra of the normalized cross-correlograms of all striatal unit pairs recorded in control and lesioned rats. Note the prominent peak in synchronized firing in the beta-frequency range in lesioned rats. ***F***, Mean power spectra of all measures of striatal BUA in controls and lesioned rats. Shaded areas in ***E*** and ***F*** show SEMs. AU, Arbitrary units.

We next examined whether and to what extent the synchronized firing of striatal neurons during cortical activation was altered by dopamine depletion (Fig. 7). The CCs of pairs of striatal neurons recorded from control rats (*n* = 180 pairs) often exhibited broad peaks that were centered at around zero lag (Fig. 7*A*). The CCs of neurons recorded in lesioned rats (*n* = 1758 pairs) often exhibited comparatively higher central peaks and more prominent side lobes with intervals of 40–50 ms (Fig. 7*B*), indicating a more prevalent beta oscillatory component in their synchronized firing. Taking into account all pairs of striatal neurons recorded in lesioned or control rats, these differences manifested as a larger central peak in the histogram of significant, positive correlations in lesioned rats (Fig. 7*C*,*D*). The histogram of significant pairs in lesioned rats also had discrete side lobes with intervals of 40–50 ms (Fig. 7*D*). Accordingly, the *z*-scores of CCs at zero-lag were significantly higher (MWUT, *p* = 0.038) for unit pairs in lesioned rats compared with pairs in controls. Finally, the power spectrum of the *z*-scored CCs of unit pairs in lesioned rats displayed a peak in the beta-frequency range (15–30 Hz), which was not present in the same measure in control animals (Fig. 7*E*). To gain insight into the activity dynamics of larger populations of striatal neurons, we next analyzed the BUA signals. The spectral power of the BUA signals at beta oscillation frequencies (15–30 Hz) was significantly higher in lesioned rats than in controls (controls, *n* = 576 probe channels; *n* = 1424 probe channels; MWUT, *p* = 1.39e-29; Fig. 7*F*), suggesting that large ensembles of striatal neurons are inappropriately recruited to the beta oscillations after dopamine depletion.

In summary, these silicon probe recordings demonstrate that the spike firing of a sizeable subpopulation of striatal neurons is phase locked to the excessive beta oscillations that emerge in cortico-basal ganglia circuits after chronic dopamine depletion. Although the firing rates of individual striatal neurons in lesioned rats are well below the frequencies of beta oscillations, the spike firing across ensembles of striatal neurons is nonetheless preferentially and excessively synchronized at 15–30 Hz.

### Some indirect pathway SPNs increase their firing rates and phase-locked firing to cortical beta oscillations after dopamine depletion

**Figure 8.**
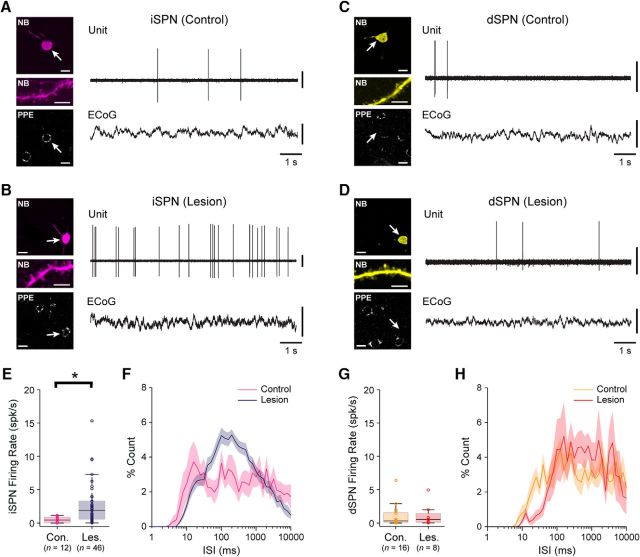
Spontaneous firing of indirect pathway SPNs and direct pathway SPNs during cortical activation in dopamine-intact and 6-OHDA-lesioned rats. ***A***, ***B***, Left side, Single-plane confocal fluorescence micrographs of indirect pathway SPNs, identified after labeling with neurobiotin (NB) by their densely spiny dendrites (middle panels), in a dopamine-intact control rat (***A***) and a lesioned rat (***B***). Both SPNs (arrows) expressed immunoreactivity for PPE, confirming them to be iSPNs (bottom). Right side, The action potentials spontaneously fired by the same identified iSPNs (unit) during cortical activation, as verified in ECoG recordings. Note that, after dopamine depletion, iSPNs tend to fire spikes more frequently. ***C***, ***D***, Micrographs of NB-labeled direct pathway SPNs in a control rat (***C***) and a lesioned rat (***D***). Neither SPN expressed immunoreactivity for PPE, identifying them as dSPNs. ***E***, Firing rates of identified iSPNs in control (Con.) and lesioned (Les.) rats. On average, iSPNs fired at significantly higher firing rates in lesioned rats. The number of SPNs included in each group is shown in parenthesis. ***F***, Mean ISI histograms for iSPNs recorded in control or lesioned rats (shaded areas show SEMs). ***G***, Firing rates of identified dSPNs in control and lesioned rats. There were no significant differences in the firing rates of dSPNs in each group. ***H***, Mean ISI histograms for dSPNs. Scale bars, ***A–D***, 20 μm; images of dendrites, 5 μm. Vertical calibration bars: ***A–D***, 0.5 mV (ECoG); 1 mV (units). *p < 0.05 (Mann–Whitney *U* test).

Having established that dopamine depletion leads to increases in the firing rates of striatal neurons during cortical activation, and that a substantial fraction of striatal neurons fire in a phase-locked manner to the abnormal beta oscillations that arise in this brain state, we next examined whether and to what extent the firing of iSPNs and dSPNs tallied with these alterations in striatal activity dynamics. We thus recorded and juxtacellularly labeled 28 SPNs in dopamine-intact control rats (*n* = 20), and 54 SPNs in 6-OHDA-lesioned rats (*n* = 15), during cortical activation (Fig. 8). In good agreement with our silicon probe data, the majority of spontaneously active SPNs in control rats, and many SPNs in lesioned rats, fired at low average rates (<2 spk/s) and with irregular patterns; neurons fired single spikes and/or brief bursts of spikes every few seconds (Fig. 8*A*,*C*,*D*). However, and in further accordance with our silicon probe data, the average firing rate of all SPNs recorded in lesioned rats (2.63 ± 0.43 spk/s; range, 0.027–15.30 spk/s) was significantly higher (MWUT, *p* = 3.38e-04) than that of SPNs in controls (0.81 ± 0.24 spk/s; range, 0.007–6.40 spk/s). Of the SPNs recorded in control rats, 12 were identified as PPE^+^ iSPNs (Fig. 8*A*) and 16 were identified as PPE^−^ dSPNs (Fig. 8*C*). Of the SPNs recorded in lesioned rats, 46 were identified as iSPNs (Fig. 8*B*) and 8 were identified as dSPNs (Fig. 8*D*). We noted that some iSPNs in lesioned rats fired at relatively high rates (>;3 spk/s; Fig. 8*B*,*E*), which were generally not matched by dSPNs (Fig. 8*D*,*G*). Accordingly, the average firing rate of iSPNs in lesioned rats (2.80 ± 0.07 spk/s) was significantly higher (MWUT, *p* = 0.0003; Fig. 8*E*) than that of iSPNs in control rats (0.49 ± 0.04 spk/s). This increase in the absolute firing rates of iSPNs was equivalent to a substantial relative increase (∼470%) in their firing rates. In stark contrast, the firing of dSPNs was relatively unaffected by dopamine depletion (Fig. 8*C*,*D*). Indeed, the average firing rate of dSPNs in lesioned rats (1.15 ± 0.20 spk/s) was not different (MWUT, *p* = 0.74; Fig. 8*G*) from that of dSPNs in control rats (1.05 ± 0.10 spk/s). It should be noted again, however, that approximately equal numbers of iSPNs and dSPNs were recorded (blinded to cell type) in control animals, whereas our sample of iSPNs in lesioned rats was six times larger than our sample of dSPNs in lesioned rats. The actual sample sizes of iSPNs and dSPNs recorded in control rats were not different from those expected from equal sampling of two populations with similar proportions of spontaneously firing neurons (Pearson's χ^2^, *p* = 0.45). However, the sample sizes of iSPNs and dSPNs recorded in lesioned rats were significantly different from those expected (Pearson's χ^2^, *p* = 2.33e-07). Thus, it is likely that, during cortical activation, a greater proportion of dSPNs is effectively quiescent after chronic dopamine depletion (see below). Together, these data demonstrate that, when the dopamine system is intact, iSPNs and dSPNs cannot be readily distinguished on the basis of their spontaneous firing rates/patterns during cortical activation *in vivo*. However, dopamine depletion was associated with increases in the firing rates of iSPNs, but not dSPNs. With our silicon probe recordings in mind, it is likely that iSPNs are the major contributors to the overall increase in firing rates that was observed in the dopamine-depleted striatal network during cortical activation.

**Figure 9.**
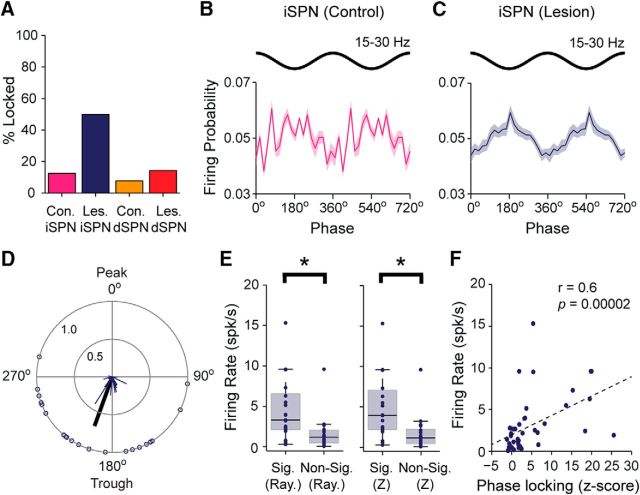
Firing of indirect pathway SPNs and direct pathway SPNs with respect to cortical beta oscillations in dopamine-intact rats and 6-OHDA-lesioned rats. ***A***, Proportions of iSPNs and dSPNs that fired in a significantly phase-locked manner to cortical beta oscillations at 15–30 Hz during the activated brain state in dopamine-intact control rats (Con.) and lesioned rats (Les.). ***B***, ***C***, Mean linear-phase histograms of the firing of all iSPNs recorded in control rats (***B***) and lesioned rats (***C***) during cortical activation. For clarity, two cortical beta-oscillation cycles are shown. Shaded areas show SEMs. Note that iSPNs in lesioned rats were more consistent in their phase locking to cortical beta oscillations. ***D***, Circular plot of the preferred firing angles of significantly phase-locked iSPNs in lesioned rats; these neurons tended to fire around the troughs of the cortical beta oscillations. ***E***, Comparison of the firing rates of iSPNs, divided according to whether their firing was significantly phase locked (Sig.) or not significantly locked (Non-Sig.) to cortical beta oscillations. The significance of phase locking was evaluated using both the Rayleigh test (Ray.; left) and a rate-normalized *z*-score (Z; right). In both cases, the iSPNs that were significantly phase locked to cortical beta oscillations had, on average, higher firing rates than the iSPNs that were not significantly locked. ***F***, The rate-normalized *z*-score of iSPN-phase locking was significantly and positively correlated with firing rate (Spearman correlation). *p < 0.05 (Mann–Whitney *U* test).

We next defined how the spike firing of iSPNs and dSPNs is temporally related to cortical beta oscillations (Fig. 9). In control rats, only a small proportion of qualifying iSPNs (*n* = 1 of 8 iSPNs) exhibited firing that was significantly phase locked to beta oscillations (Fig. 9*A*). As a group, iSPNs in control rats showed a correspondingly weak tendency to fire around the troughs of the beta oscillations (Fig. 9*B*). Dopamine depletion had a substantial effect on the phase-locked firing of iSPNs. In lesioned rats, half of the qualifying iSPNs (*n* = 22 of 44) exhibited firing that was significantly phase locked to beta oscillations (Fig. 9*A*). The observed proportion of iSPNs with firing that was phase locked to beta oscillations in lesioned rats was significantly different (i.e., much larger) than the proportion expected from iSPN recordings in controls (Pearson's χ^2^, *p* = 5.42e-14). As a group, iSPNs in lesioned rats showed a clear tendency to discharge around the troughs of the cortical beta oscillations (95% of significantly locked iSPNs had preferred angles between >;90° and <270°; mean angle, 199.0 ± 9.6°; Fig. 9*C*,*D*), a phase preference that was similar to that exhibited by many striatal units we recorded with silicon probes in lesioned rats (Fig. 6*C*,*E*). In contrast to the scenario for iSPNs, dopamine depletion had little impact on the phase-locked firing of dSPNs. Indeed, only small proportions of the dSPNs in control and lesioned rats exhibited firing that was significantly phase locked to beta oscillations (*n* = 1 of 13 dSPNs in controls; *n* = 1 of 7 dSPNs in lesioned rats; Fig. 9*A*). Accordingly, dopamine depletion did not change the proportion of dSPNs that phase locked their firing to cortical beta oscillations (binomial test, *p* = 0.85). Group analyses suggested that dSPNs had no obvious beta-phase preference in their firing, regardless of whether they were recorded in control or lesioned rats.

The data above show that iSPNs in lesioned animals have, in relative terms, high firing rates and a strong tendency to phase lock their firing to ongoing beta oscillations. We next examined whether these two firing properties were related. First, we tested whether the iSPNs that fired in a significantly phase-locked manner to beta oscillations had higher firing rates than iSPNs that were not phase locked. When using Rayleigh's uniformity test to define significantly phase-locked firing, the average firing rate of iSPNs that were phase locked to beta oscillations (4.58 ± 0.18 spk/s) was more than twice that of iSPNs that were not phase locked (1.60 ± 0.08 spk/s; MWUT, *p* = 0.002, Fig. 9*E*). As the significance of the Rayleigh test could potentially be influenced by the number of spikes fired, we recomputed this comparison using the phase-locking *z*-score based on surrogates of shuffled ISIs. Using this rate-normalized measure, the difference in the firing rates of iSPNs that were and were not significantly phase locked was confirmed with more robust statistics (MWUT, *p* = 8.35e-04; Fig. 9*E*). Moreover, the phase-locking *z*-score was significantly and positively correlated with the firing rate of iSPNs (Spearman correlation, *r* = 0.6, *p* = 1.96e-05; Fig. 9*F*). Together, these data show that iSPNs that fired in a significantly phase-locked manner to abnormal beta oscillations were more active than other SPNs.

**Figure 10.**
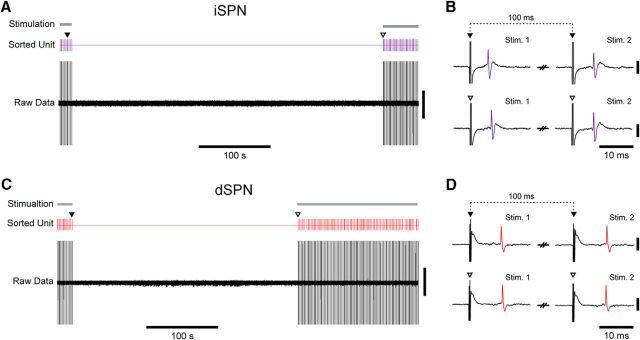
Some indirect pathway SPNs and direct pathway SPNs are quiescent during the activated brain state in 6-OHDA-lesioned rats. ***A***, Quiescent iSPN recorded during the activated brain state in a lesioned rat. The iSPN was revealed by paired electrical stimulation of the motor cortex (periods of stimulation are indicated by gray bars). Raw data show unsorted unit activity and stimulus artifacts in striatum (signals truncated for clarity). “Sorted Unit” indicates the occurrence of spike firing by the individual identified iSPN. Note that, in the absence of cortical stimulation, the iSPN does not spontaneously fire any spikes for an extended period of recording (hundreds of seconds). The iSPN was thus defined as effectively quiescent. After the recording of spontaneous unit activity, cortical stimulation was resumed and the iSPN was correspondingly driven to fire spikes. Black and white triangles indicate epochs that are shown in ***B*** at higher resolution. ***B***, Representative traces of the short-latency responses of the same iSPN to the paired cortical stimulation (Stim. 1, Stim. 2; 100 ms interval between each stimulus) delivered before the extended period of quiescence (top traces, stimulus delivery marked by black triangles) and after the period of quiescence (bottom traces, stimulus delivery marked by white triangles). Stimulation-evoked spikes are highlighted in violet; note that the spikes are of a similar shape, magnitude, and duration in the stimulation periods before and after the registration of spontaneous firing, indicating the iSPN was proximate to the recording electrode throughout. ***C***, ***D***, Same as in ***A*** and ***B*** but for a quiescent dSPN recorded during the activated brain state in a lesioned rat. Stimulation-evoked spikes of the dSPN are highlighted in red. Vertical calibration bars: ***A–D***, 1 mV.

Our experiments show that, after dopamine depletion, many iSPNs exhibit abnormally increased rates of spontaneous firing during cortical activation. However, our recordings do not rule out the possibility that other iSPNs are effectively quiescent in this brain state. Moreover, because our sample of spontaneously firing iSPNs was substantially larger than our sample of spontaneously firing dSPNs during cortical activation in lesioned rats, it is also likely that a greater proportion of dSPNs are effectively quiescent under these conditions. To address both of these issues, we used focal electrical stimulation of the motor cortex to test for the presence of SPNs in lesioned rats that were effectively quiescent (Mallet et al., 2005, 2006; Ballion et al., 2009; Kita and Kita, 2011; Escande et al., 2016), defined here as SPNs that did not spontaneously fire or at least exhibited very low rates of spontaneous firing (<0.03 spk/s) during long-duration recording epochs (300–450 s) without cortical stimulation (Fig. 10). The paired electrical stimuli were delivered to motor cortex while slowly advancing the glass electrode through the ipsilateral dorsal striatum; upon encountering a single unit that fired action potentials at short and consistent latencies in response to the cortical stimulation, the electrode movement and cortical stimulation were stopped (Fig. 10*A*,*C*). After recording the spontaneous unit activity for 300–450 s, the cortical stimulation was resumed to ensure that the same single unit was still proximate to the recording electrode (Fig. 10*B*,*D*) before juxtacellularly labeling it with neurobiotin. Revealing effectively quiescent neurons in this manner, we recorded the spontaneous firing of 15 stimulation-responsive SPNs (6 PPE^+^ iSPNs and 9 PPE^−^ dSPNs) in lesioned rats during cortical activation. Three of the iSPNs did not fire at all in the absence of cortical stimulation (Fig. 10*A*,*B*), while the other three iSPNs fired at very low rates (range, 0.013–0.022 spk/s). Six of the dSPNs did not fire at all in the absence of cortical stimulation (Fig. 10*C*,*D*), while the other three dSPNs fired at very low rates (range, 0.002–0.007 spk/s). These data confirm that, after dopamine depletion, some iSPNs and dSPNs are effectively quiescent in this brain state. Together, these observations further suggest that a greater proportion of dSPNs are effectively quiescent under these conditions.

In summary, these recordings of individual, identified iSPNs and dSPNs during cortical activation demonstrate that dopamine depletion is associated with substantial increases in the firing rate and beta oscillation-coupled firing of iSPNs but not dSPNs. These dramatic alterations in the firing of iSPNs are restricted to a subpopulation of these neurons, an observation further emphasized by the fact that other iSPNs are effectively quiescent during cortical activation in lesioned rats. With our silicon probe recordings in mind, it is likely that iSPNs are the major contributors to the large subpopulation of striatal neurons that fire in a phase-locked manner to the excessive beta oscillations that emerge in cortico-basal ganglia circuits after chronic dopamine depletion.

### Firing properties of identified spiny projection neurons in relation to their distributions in striatal territories and neurochemical compartments

**Figure 11.**
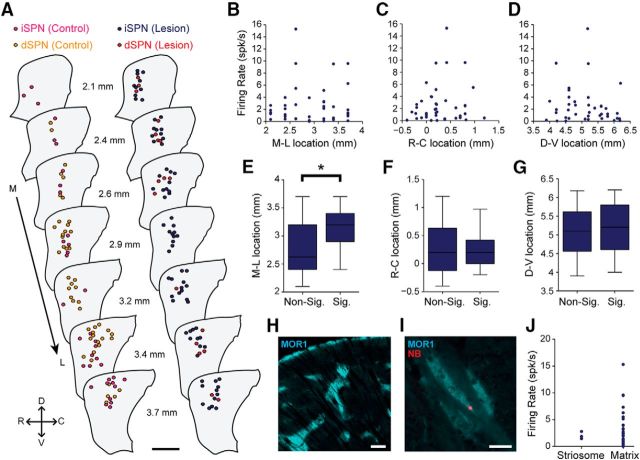
Distributions of recorded and identified SPNs in dopamine-intact rats and 6-OHDA-lesioned rats. ***A***, Locations of PPE^+^ iSPNs and PPE^−^ dSPNs recorded in dopamine-intact control rats (left) and lesioned rats (right), as mapped on seven parasagittal sections of dorsal striatum (from 2.1 to 3.7 mm lateral of Bregma). M, medial; L, lateral; R, rostral; C, caudal; D, dorsal; V, ventral. Each circle represents either an iSPN (magenta, recorded in controls; dark blue, in lesioned rats) or a dSPN (orange, recorded in controls; red, in lesioned rats). ***B***–***D***, Firing rate of each iSPN recorded in lesioned rats during the activated brain state plotted against its location in the mediolateral (***B***), rostrocaudal (***C***), and dorsoventral (***D***) axes of striatum. Locations are reported with respect to Bregma. The firing rates of these iSPNs were not significantly correlated with their locations along any axis. ***E***–***G***, Comparisons of the mediolateral (***E***), rostrocaudal (***F***), and dorsolateral (***G***) locations of the same iSPNs as a function of whether their firing was significantly phase locked (Sig.) or not (Non-Sig.) to cortical beta oscillations. The iSPNs with significantly phase-locked firing were located more lateral than iSPNs that were not phase locked (***E***). ***H***, Enriched immunoreactivity for MOR1 reveals the striosome compartments of the dorsolateral striatum. ***I***, Example of a neurobiotin (NB)-labeled neuron with a soma located within a striosome expressing high levels of MOR1. ***J***, Firing rates of all iSPNs recorded during cortical activation in lesioned rats as a function of their locations in striosomes or matrix. The firing rates of the striosome iSPNs were toward the lower end of the range of firing rates of matrix iSPNs. Scale bars: ***A***, 1.5 mm; ***H***, ***I***, 200 μm. *p < 0.05 (Mann–Whitney *U* test).

Some of the differences in the firing properties of iSPNs and dSPNs might have arisen through a subtle bias in the striatal territories in which they were sampled. To explore this, we mapped the locations of the recorded and identified iSPNs and dSPNs onto a series of seven parasagittal sections of striatum, ranging from 2.1 to 3.7 mm lateral of Bregma (Paxinos and Watson, 2007; Fig. 11*A*; one iSPN recorded in control animals was located ∼1.6 mm lateral of Bregma and is not shown). The maps revealed that most SPNs were located in the “dorsal half” and “lateral half” of striatum (Fig. 11*A*), territories that receive particularly dense glutamatergic inputs from the sensorimotor cortex (McGeorge and Faull, 1989; Reep et al., 2003). Importantly, the maps also showed that not only were SPNs recorded in comparable areas of striatum in the control and lesioned rats, but also that there was extensive overlap in the locations of iSPNs and dSPNs (Fig. 11*A*), arguing against the possibility of a strong or systematic spatial bias in the sampling of these cell types. However, this does not rule out the possibility that, within a group comprised of one cell type, location may predict some firing properties. We examined whether variations in the firing rates and levels of beta oscillation-coupled firing across iSPNs during cortical activation in lesioned rats (Figs. 8, 9) were related to their recording locations within dorsal striatum. The firing rates of these iSPNs were not significantly correlated with their positioning along the mediolateral, rostrocaudal, or dorsoventral axes of striatum (Fig. 11*B–D*). However, the locations of iSPNs with firing that was significantly phase locked to cortical beta oscillations were on average more lateral than the locations of the iSPNs that were not phase locked (MWUT, *p* = 0.03; Fig. 11*E*); there was no difference along the other two axes (Fig. 11*F*,*G*). These data suggest that the prevalence or size of the subpopulation of iSPNs that fire in time with the abnormal beta oscillations partly depends on (the relative laterality of) the striatal territory in which they are located.

The striatum is not only dichotomously organized into distinct output pathways, but also into striosome/patch and matrix compartments (Graybiel and Ragsdale, 1978; Gerfen, 1984). The *in vivo* electrophysiological properties of identified SPNs that are located within neurochemically-defined striosomes are unclear. The present study included recordings of 13 identified SPNs with somata that were located within striosomes, defined as circumscribed areas of striatal neuropil that displayed highly enriched immunoreactivity for μ-opioid receptors (Fig. 11*H*,*I*). Of these 13 striosome SPNs, 3 were iSPNs recorded in control rats, 4 were dSPNs recorded in control rats, 4 were iSPNs recorded in lesioned rats, and 2 were dSPNs recorded in lesioned rats. These small sample sizes are not unexpected given that striosomes make up only ∼15% of the volume of the dorsal striatum of rats (Crittenden and Graybiel, 2011). Qualitative assessments of the firing rates and patterns of striosome SPNs suggested that, regardless of brain state, their activity was comparable to that of SPNs located in the matrix compartment. As such, striosome SPNs and matrix SPNs were pooled for all the analyses of identified SPNs detailed above. Focusing on iSPNs recorded during cortical activation in lesioned animals, it was, however, evident that the firing rates of striosome iSPNs (*n* = 3) were at the lower end of the range of firing rates exhibited by matrix iSPNs (Fig. 11*J*). None of these striosome iSPNs fired in a significantly phase-locked manner with cortical beta oscillations. Although the small sample sizes of striosome SPNs precluded detailed statistical comparisons, these data collectively suggest that, in many respects, the spontaneous firing rates/patterns of striosome SPNs *in vivo* are within the ranges exhibited by matrix SPNs *in vivo*. However, with respect to our silicon probe recordings, it is likely that matrix iSPNs and not striosome iSPNs are the major contributors to the large subpopulation of striatal neurons that fire at relatively high rates and in a phase-locked manner to the excessive beta oscillations arising after chronic dopamine depletion.

### Neuronal ensembles enriched for putatively-classified indirect pathway SPNs selectively synchronize their firing at beta frequencies after dopamine depletion

**Figure 12.**
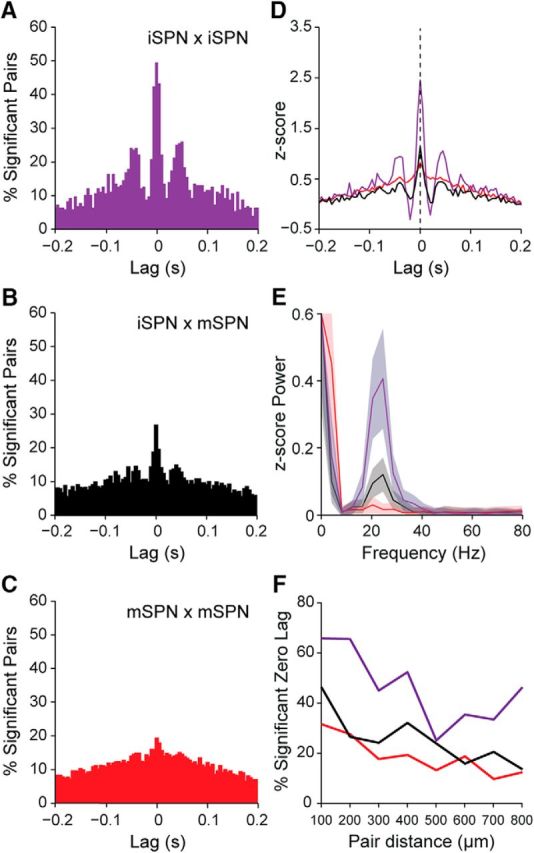
Dopamine depletion is associated with a selective increase in the synchronization of firing in neuronal ensembles enriched for putatively-classified indirect pathway SPNs. ***A***–***C***, Histograms of significant, positive correlations in spike firing for pairs of putative iSPNs (***A***, in violet), for pairs consisting of one putative iSPN and one putative mSPN (***B***, in black), and for pairs of putative mSPNs (***C***, in red). Single units recorded with silicon probes during the activated brain state in lesioned rats were classified as putative iSPNs when their firing properties met the criteria that enrich ensembles for iSPNs, whereas the single units not meeting these criteria were classified as putative mSPNs. Cross-correlograms between pairs of putative SPNs were calculated and converted to a *z*-score using ISI-shuffled surrogate spike trains with identical mean firing rates and ISI distributions to the real data. Also see Fig. 12-1. Note that only the pairs of putative iSPNs (***A***) had a strong beta-oscillatory component in their synchronized firing, as indicated by a relatively high central peak around zero lag as well as prominent side lobes with intervals of 40–50 ms. ***D***, ***E***, Normalized (*z*-scored) cross-correlograms (***D***), and their corresponding power spectra (***E***), for the three pair types in ***A–C***. Ensembles enriched for putative iSPNs (purple) exhibited the most prevalent synchronization of spike firing at beta-oscillation frequencies. ***F***, Mean percentage of significant correlations (at zero lag) in the three pair types as a function of the spatial separation between the paired units. There were approximately twice as many synchronized pairs of putative iSPNs than other pair types for most distances of up to 800 μm. Data in ***E*** are means, with shaded areas indicating 99% confidence intervals. Colors in ***D–F*** are as in ***A–C***.

Our silicon probe recordings showed that a subpopulation of striatal neurons preferentially and excessively synchronizes their spike firing at beta-oscillation frequencies after dopamine depletion (Fig. 7). Our recordings of individual identified projection neurons suggested that iSPNs are the major contributors to this subpopulation. In a penultimate set of analyses (Fig. 12), we interrogated the single-unit activities recorded with silicon probes in the context of the confirmed firing properties of iSPNs and dSPNs. Taking the probe data recorded in lesioned rats during cortical activation, we first classified the single units according to whether or not their firing properties matched those of many spontaneously firing iSPNs in lesioned rats during cortical activation [i.e., firing rates of 2–20 spk/s, and significantly phase-locked firing to cortical beta oscillation troughs (phase angles of between >;90° and <270°)]. The population of single units that met these combined criteria was likely to be highly enriched for iSPNs. Indeed, 94% of identified SPNs in lesioned rats meeting these criteria were iSPNs (Fig. 8). We thus designated such single units as “putative iSPNs”. The other single units that did not meet these combined criteria were designated as “putative mixed SPNs” (mSPNs) to reflect the fact that identified SPNs that fired at rates <2 spk/s and/or that did not fire phase locked to beta oscillation troughs were a more even mixture of dSPNs and iSPNs. We then recomputed cross-correlograms for pairs of putative iSPNs (*n* = 208), for pairs consisting of one putative iSPN and one putative mSPN (*n* = 482), and for pairs of putative mSPNs (*n* = 1098). The histogram of significant positive correlations for pairs of putative iSPNs had a relatively high central peak (around zero lag) and prominent side lobes with intervals of 40–50 ms (Fig. 12*A*), indicating a prevalent beta oscillatory component in their synchronized firing. However, the histograms of significant iSPN/mSPN pairs and significant mSPN/mSPN pairs exhibited comparatively small central peaks with smaller or no apparent side lobes (Fig. 12*B*,*C*). Accordingly, 50% of the putative iSPN/iSPN pairs were significantly correlated at zero lag, whereas <30% of iSPN/mSPN pairs and <20% of mSPN/mSPN pairs were correlated (Fig. 12*A–C*). The *z*-scored CCs for the three pair types mirrored the histograms of significant correlations; only the CC for putative iSPN/iSPN pairs had a large central peak and prominent side lobes with intervals of 40–50 ms (Fig. 12*D*). The *z*-scores of CCs at zero lag were significantly different among the three pair types (Kruskal–Wallis ANOVA, χ^2^ = 107, *p* = 2.80e-27), with those for putative iSPN/iSPN pairs (2.45 ± 0.18) being significantly greater on average than those of putative iSPN/mSPN pairs (1.17 ± 0.08) and of putative mSPN/mSPN pairs (0.82 ± 0.06; *post hoc* Dunn's tests). The *z*-scores of putative iSPN/mSPN pairs were also significantly greater on average than those of putative mSPN/mSPN pairs. The power spectra of the CC *z*-scores of putative iSPN/iSPN pairs displayed a prominent peak in the beta-frequency range (15–30 Hz), which contained significantly more power than the minor peaks of iSPN/mSPN pairs and mSPN/mSPN pairs (Kruskal–Wallis ANOVA, *p* = 1.90e-57, χ^2^ = 265, *post hoc* Dunn's tests; Fig. 12*E*). Last, we analyzed the prevalence of significant correlations at zero lag in iSPN/iSPN pairs, iSPN/mSPN pairs, and mSPN/mSPN pairs as a function of the spatial separation between the paired units (with separation between paired units being defined as the distance between the silicon probe contacts on which they were recorded). There were approximately twice as many synchronized iSPN/iSPN pairs as iSPN/mSPN pairs or mSPN/mSPN pairs for most distances of up to 800 μm (Fig. 12*F*). It is important to note that, because cross-correlograms were calculated and converted to a *z*-score using ISI-shuffled surrogate spike trains that had mean firing rates and ISI distributions identical to the real data (see Materials and Methods), the increased correlations observed for pairs of putative iSPNs were not simply the result of the higher mean firing rates of these units. In a final set of analyses (Fig. 12-1), we tested whether this selective increase in the synchronized oscillatory firing of putative iSPNs was a result of classifying single units according to the characteristics of their phase-locked firing with respect to cortical beta oscillations. To address this, we recomputed cross-correlograms for the three pair types using surrogate spike trains that had the same mean firing rates and the same phase distributions as the real data. This analysis using beta phase-controlled surrogates produced results that were similar in all respects to those obtained using ISI-shuffled surrogates (compare Figs. 12, 12-1), suggesting that any shared phase-locking profiles could not alone account for the preferential rhythmic synchronization of ensembles enriched for putative iSPNs. Together, both sets of analyses demonstrated that differences in correlations were not dependent on the higher firing rates of units in ensembles enriched for putative iSPNs.

10.1523/JNEUROSCI.0658-17.2017.f12-1 Figure 12-1 Dopamine depletion is associated with a selective increase in the synchronization of firing in neuronal ensembles enriched for putatively-classified indirect pathway SPNs. *A*–*C*, Histograms of significant, positive correlations in spike firing for pairs of putative iSPNs (*A*, in violet), for pairs consisting of one putative iSPN and one putative mSPN (*B*, in black), and for pairs of putative mSPNs (*C*, in red). Cross-correlograms between pairs of putative SPNs were calculated and converted to a z-score using beta-phase-shuffled surrogate spike trains with identical mean firing rates and phase distributions (with respect to cortical beta oscillations) to the real data. *D*, *E*, Normalized (z-scored) cross-correlograms *(D*), and their corresponding power spectra (*E*), for the three pair types in *A*–*C*. Ensembles enriched for putative iSPNs (purple) exhibited the most prevalent synchronization of spike firing at beta-oscillation frequencies. *F*, Mean percentage of significant correlations (at zero lag) in the three pair types as a function of the spatial separation between the paired units. Note that the results here are similar in all respects to those obtained using ISI-shuffled surrogate spike trains. Data in *E* are means, with shaded areas indicating 99% confidence intervals. Colors in *D*–*F* are as in *A*–*C*. Download Figure 12-1, TIF file

In summary, these silicon probe recordings of putatively classified SPNs suggest that a subpopulation of iSPNs, distributed across large areas of striatum, preferentially and excessively synchronize their spike firing at beta-oscillation frequencies after chronic dopamine depletion. This synchronized firing is highly selective, such that it is not prevalent in ensembles containing other SPNs. The firing of a subpopulation of iSPNs is thus likely to underpin the abnormal beta-frequency synchronized output from striatum that arises after dopamine depletion.

## Discussion

Here, we shed light on how the chronic depletion of dopamine alters the temporal dynamics of electrical activity in the dorsal striatum *in vivo*. Our data converge to demonstrate a cell type-selective entrainment and synchronization of striatal neuron firing during the abnormal network oscillations that arise in cortical–basal ganglia circuits in parkinsonism. We used male rats, and care should be taken in extrapolating our results to females.

### Dopamine depletion alters the firing rates of spiny projection neurons

Using silicon probes, we sampled unit activity in the striatum of dopamine-intact and 6-OHDA-lesioned rats during two well defined brain states, cortical SWA and activation. Our complementary recordings of identified neurons strongly suggest that SPNs (of the matrix compartment) constituted the vast majority of single units sampled with silicon probes. Altogether, our data indicate that, regardless of brain state, chronic dopamine depletion is associated with significant escalations in striatal net output. Similar conclusions have been previously drawn from recordings of putatively classified SPNs made in anesthetized or awake rats (Kish et al., 1999; Chen et al., 2001; Tseng et al., 2001; Kita and Kita, 2011; Zold et al., 2012), and in awake monkeys (Liang et al., 2008) and humans (Singh et al., 2016). Increased striatal net output in parkinsonism also validates predictions from several computational models (Kumar et al., 2011; McCarthy et al., 2011; Damodaran et al., 2015). As anticipated (Nevado-Holgado et al., 2014), the average firing rate of striatal neurons approximately doubled during transitions from SWA to activation, which is particularly relevant because the latter is more akin to brain states observed in awake animals. Many of the striatal neurons we recorded had low firing rates (i.e., 0.1–3.0 spk/s), which are consistent with those of identified and putatively classified SPNs recorded in awake rats and monkeys at rest (Chen et al., 2001; Mahon et al., 2006; Kita and Kita, 2011; Isomura et al., 2013; Deffains et al., 2016). There were, however, notable exceptions (see below).

A key innovation here is the *in vivo* definition of the firing of identified SPNs of the direct or indirect pathways. Our recordings of dSPNs and iSPNs revealed that, during SWA, dopamine depletion is associated with increases in the firing rates of both cell types. However, during cortical activation, dopamine depletion was associated with an increase in the firing rates of iSPNs, but not dSPNs. These and other data reinforce the importance of quantifying striatal activity within the context of defined brain states (Magill et al., 2006; Sharott et al., 2012; Zold et al., 2012). Increases in SPN firing rates were modest in absolute terms but large in relative terms (170–470%). Almost all SPNs firing at comparatively high rates (3–20 spk/s) in lesioned animals were iSPNs. These recordings are of special value for appraising a core prediction of the direct/indirect pathways model, namely that dopamine loss imbalances the firing rates of dSPNs and iSPNs (i.e., they become hypoactive and hyperactive, respectively). To our knowledge, only one previous study (of a small set of identified SPNs recorded during SWA in anesthetized rats) has provided direct, real-time evidence of imbalanced dSPN/iSPN firing rates *in vivo* after dopamine depletion (Mallet et al., 2006). Interestingly, the same study reported that dSPNs and iSPNs were consistently “silenced” during cortical activation in 6-OHDA-lesioned rats (Mallet et al., 2006), which would not support the notion of imbalanced pathways per se. Considering the prevalence and firing rates of the spontaneously active dSPNs and iSPNs we recorded, we can conclude that, first, the predicted imbalance in striatal output pathways is present but relatively slight during SWA, and second, this imbalance is exacerbated during cortical activation. We demonstrate that some dSPNs and iSPNs in the dopamine-depleted striatum are effectively quiescent during activation; a greater proportion of dSPNs is likely quiescent under these conditions. This “disproportionate quiescence” of dSPNs and iSPNs could further aggravate the imbalance in striatal output pathways arising from dopamine depletion.

### Impact of dopamine depletion on the oscillatory synchronization of striatal activity

Our data highlight that chronic dopamine depletion not only alters the firing rates of SPNs but also the rhythmic synchronization of SPN firing. Several features of the disturbed striatal activity dynamics relating to the slow (∼1 Hz) oscillations prevalent during SWA also generalized to the excessive beta-frequency (15–30 Hz) oscillations that emerged during activated brain states. Thus, in both cases, our silicon probe recordings suggested that SPNs in lesioned rats have higher incidences of phase-locked firing to ongoing cortical oscillations, and that SPN ensembles have higher levels of oscillatory, synchronized firing. Our recordings of identified neurons established that iSPNs are particularly prone to being recruited to abnormal network oscillations. Of special note, our data collectively argue that a population of iSPNs, distributed across large areas of dorsal striatum, preferentially and excessively synchronize their firing at beta oscillation frequencies after dopamine depletion. Because the firing rates of individual SPNs are almost always lower than beta frequencies, a given SPN discharges on a minority of beta oscillation cycles. This “incomplete engagement” of iSPNs could underlie their seemingly irregular firing patterns. However, the firing of some iSPNs is nevertheless precisely organized in time; their firing is synchronized (with small lags) and phase locked (to limited angles). Thus, after dopamine depletion, population-level beta oscillations inappropriately emerge and are output from networks of iSPNs exhibiting low firing rates and irregular (non-oscillatory) firing patterns; this scenario has been envisaged in some pertinent theoretical studies (McCarthy et al., 2011; Damodaran et al., 2015; Corbit et al., 2016). Importantly, there was a positive correlation between the firing rates and strengths of phase-locked firing of iSPNs. Thus, in the dopamine-depleted striatum, excessive firing rates are linked to excessive oscillatory synchronization of firing, a notion reinforced by our observation that dSPNs are not hyperactive and are not synchronized to large extents. Collectively, our data argue for extending the influential concept of a pathological imbalance in striatal output pathways from firing rates to the organization of rhythmic correlated firing.

### Parkinsonian beta oscillations in cortico-basal ganglia circuits

Our results provide important new insights into the neuronal substrates of the excessive beta oscillations that emerge throughout cortico-basal ganglia circuits in parkinsonism. We demonstrate that, after dopamine depletion, there is an abnormal beta frequency-synchronized output from striatum. This is likely underpinned by the firing of a population of iSPNs. By definition, all iSPNs innervate the GPe. After dopamine depletion, the oscillatory firing of GPe neurons also becomes selectively and excessively synchronized at beta frequencies (Mallet et al., 2008a). In this abnormal network state, prototypic GPe neurons, the most numerous GABAergic pallidal cell type, are also hypoactive and anomalously fire around the peaks of cortical beta oscillations (Mallet et al., 2008a; Abdi et al., 2015). Given that GABAergic iSPNs in the dopamine-depleted striatum are hyperactive, excessively synchronized, and tend to discharge around the troughs of cortical beta oscillations, they are prime candidates for shaping this aberrant “anti-phase” synchronized firing of prototypic GPe neurons, in accordance with computational modeling (Nevado-Holgado et al., 2014). As a population, prototypic GPe neurons innervate the GPe and all other BG nuclei (Mallet et al., 2012; Abdi et al., 2015); they are well placed to orchestrate and propagate exaggerated beta oscillations in BG circuits. The “feed-forward” consequences of the beta-synchronized outputs of iSPNs could thus extend far beyond GPe. In theory, the positive “feed-back” loop between GPe and striatum could further promote the rhythmic synchronization of firing (Corbit et al., 2016). Conversely, work in dopamine-depleted monkeys has emphasized that the GPe–STN network, with its specialized cortical inputs, has primacy in generating parkinsonian beta oscillations (Nambu and Tachibana, 2014; Deffains et al., 2016). In support of this, striatal LFPs recorded in these monkeys contain exaggerated oscillations at 8–15 Hz, but putatively classified SPNs are not hyperactive and do not overtly synchronize their firing at these frequencies (Deffains et al., 2016). These discrepancies in striatal activity dynamics could stem from differences in species, methods of depleting dopamine, and/or recording conditions (including cell identities and locations). Importantly, our data do not invalidate the idea that the GPe–STN network is critical for parkinsonian beta oscillations, but rather, support the concept that striatum, or more specifically, a population of iSPNs, has a central and complementary role to play.

The excessively synchronized firing of SPNs during parkinsonian beta oscillations is most prominent in ensembles enriched for iSPNs. Given that dSPNs are neither hyperactive nor excessively synchronized, they might play somewhat minor roles in orchestrating the beta oscillations expressed in their principal extrinsic targets, the output nuclei of the BG (Brown et al., 2001; Avila et al., 2010). It follows that rhythmic entrainment of neuronal activity in BG output nuclei could instead be mediated by synchronized oscillatory inputs from STN and/or GPe (Nambu and Tachibana, 2014; Deffains et al., 2016), thus reiterating the importance of disturbed activity dynamics along the whole indirect pathway in parkinsonism (Moran et al., 2011). We conclude that iSPNs, the cells of origin of the indirect pathway, could engage both monosynaptic and polysynaptic substrates to influence the generation and/or dissemination of parkinsonian beta oscillations throughout the BG.
