# Supplementary figures and images for: A Population of Indirect Pathway Striatal Projection Neurons Is Selectively Entrained to Parkinsonian Beta Oscillations
Source: J Neurosci. 2017 Oct 11;37(41):9977–98. doi: 10.1523/JNEUROSCI.0658-17.2017 (PMC5637121; doi:10.1523/JNEUROSCI.0658-17.2017)

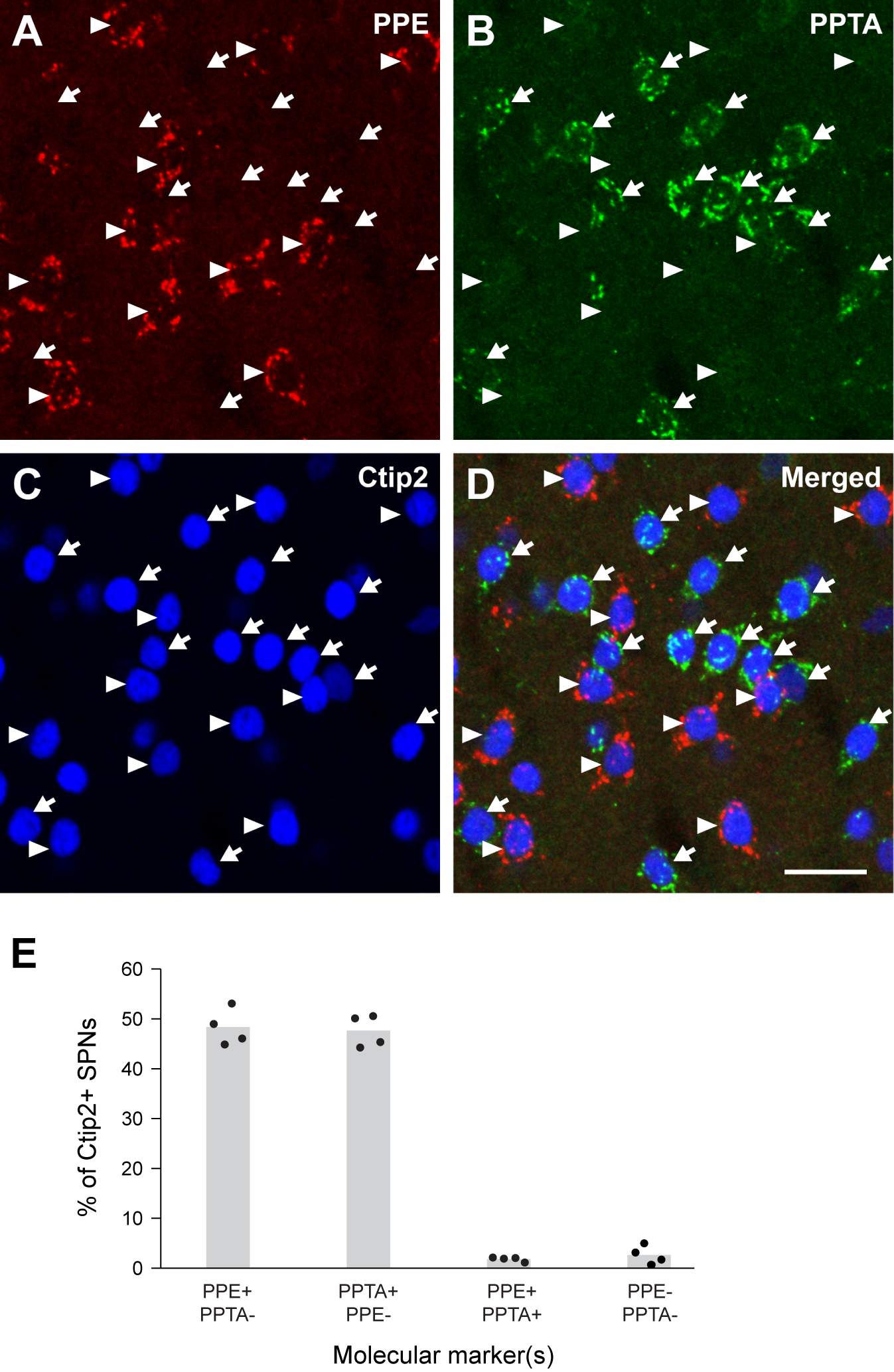

Supplement: Figure 3-1 [file zns999170117so1.tif]

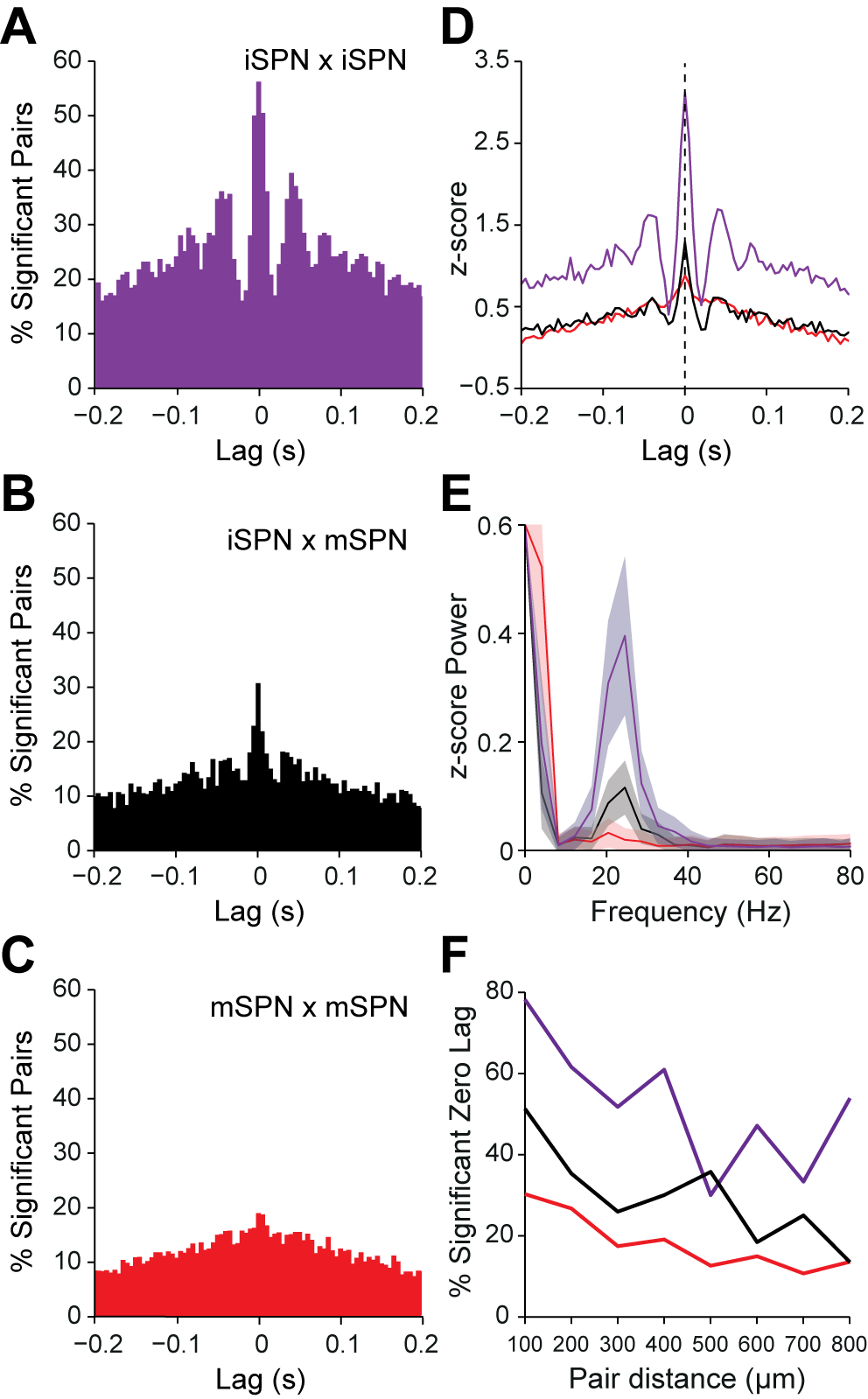

Supplement: Figure 12-1 [file zns999170117so2.tif]
